# Moderate gamma irradiation enhances the biostimulant activity of humic acid in tomato: Integrated effects on growth, physiology, yield, and fruit quality

**DOI:** 10.1371/journal.pone.0357270

**Published:** 2026-09-21

**Authors:** Mehdi Nourzadeh Hadad, Mojtaba Kordrostami

**Affiliations:** Nuclear Agriculture Research School, Nuclear Science and Technology Research Institute (NSTRI), Karaj, Iran; Amity University Mumbai, INDIA

## Abstract

Humic acid is widely used as a plant biostimulant, but its effectiveness depends strongly on its physicochemical characteristics and application rate. Gamma irradiation may modify the structure and biological activity of humic substances, although its effects on horticultural crops remain insufficiently characterized. This study evaluated the effects of non-irradiated and gamma-irradiated humic acid on the growth, physiological performance, mineral nutrition, antioxidant status, yield, and fruit quality of tomato (*Solanum lycopersicum* L.). The humic-acid treatments were arranged factorially using three irradiation doses—0, 2, and 5 kGy—and two application rates—2.5 and 5.0 kg ha^−1^—together with an untreated control. Gamma irradiation dose and humic acid application rate significantly affected most measured traits, whereas their interaction was significant only for total marketable yield. Humic acid irradiated at 2 kGy and applied at 5.0 kg ha^−1^ produced the greatest plant height, leaf area, root dry weight, chlorophyll index, net photosynthetic rate, leaf nitrogen, phosphorus and potassium concentrations, antioxidant activity, fruit number, marketable yield, total soluble solids, vitamin C, and lycopene content. Increasing the humic acid rate from 2.5 to 5.0 kg ha^−1^ significantly increased marketable yield when the humic acid was non-irradiated or irradiated at 2 kGy, but not when irradiated at 5 kGy. Treatment-adjusted correlation analysis linked canopy development and photosynthetic performance with fruit production and marketable yield, while days to first harvest was negatively associated with major growth and productivity traits. Hierarchical clustering and principal component analysis separated the treatment comprising humic acid irradiated at 2 kGy and applied at 5.0 kg ha^−1^ from the remaining treatments because of its consistently favorable multivariate profile. An ensemble multi-criteria decision-making framework integrating TOPSIS, VIKOR, WASPAS, and EDAS also ranked this treatment first under alternative criterion-weighting scenarios. These results indicate that moderate gamma irradiation can enhance humic acid biostimulant activity, whereas a higher irradiation dose may reduce the benefits obtained from increasing the application rate.

## Introduction

Tomato (*Solanum lycopersicum* L.) is one of the most widely cultivated horticultural crops and an important source of vitamins, minerals, carotenoids, and other health-promoting compounds in the human diet [1]. Sustaining tomato productivity and fruit quality has become increasingly challenging because intensive cultivation can reduce soil fertility, impair nutrient-use efficiency, and increase dependence on mineral fertilizers [2,3]. These constraints have stimulated interest in plant biostimulants that improve crop performance through physiological and biochemical regulation rather than through direct nutrient supply alone.

Humic substances are heterogeneous organic materials formed during the transformation of plant and microbial residues. Humic acid, one of their principal bioactive fractions, is widely used in horticulture because it can improve rhizosphere properties, nutrient availability, root development, photosynthetic activity, and crop productivity [4–6]. Its effects have been associated with enhanced plasma-membrane H ⁺ -ATPase activity, stimulation of root branching, improved ion transport, regulation of primary metabolism, and activation of hormone-like signaling pathways [7–9]. In tomato, humic acid application has been reported to increase vegetative growth, chlorophyll content, nutrient accumulation, fruit set, yield, and selected fruit-quality attributes [5,10].

Despite these benefits, humic acid performance is often inconsistent across crops and production systems. Its biological activity depends on several factors, including source material, molecular-size distribution, functional-group composition, solubility, application method, environmental conditions, and applied concentration [6,11]. Humic materials with different structural characteristics may interact differently with roots, nutrients, membranes, and signaling pathways. Consequently, strategies capable of modifying humic acid structure without introducing toxic chemical residues may improve the reproducibility and magnitude of its biostimulant effects.

Ionizing radiation offers a potentially useful approach for modifying complex organic materials. Gamma irradiation can induce bond cleavage, oxidation, molecular fragmentation, and redistribution of oxygen-containing functional groups through direct energy deposition and indirect reactions involving radiolytically generated reactive species [12,13]. In humic substances, these processes may alter molecular-weight distribution, solubility, complexation capacity, and the accessibility of carboxylic and phenolic groups [13,14]. Such changes could influence the interaction of humic acid with mineral ions, root surfaces, and cellular signaling systems. Gamma irradiation also has the practical advantage of producing physicochemical modification without leaving conventional chemical residues.

The effects of irradiation are expected to be dose dependent. Moderate irradiation may partially fragment large and less soluble humic structures, potentially increasing the availability of smaller and more reactive fractions. Excessive irradiation, however, may overoxidize or degrade biologically active components and thereby reduce the desired stimulatory effect [13–15]. The biological outcome may also depend on the humic acid application rate. A modified humic material may provide a favorable response at one application rate but become ineffective or physiologically imbalanced at another. Therefore, irradiation dose and humic acid rate should be examined factorially rather than as isolated treatment factors. Gamma irradiation can modify humic substances through competing processes of molecular fragmentation, oxidation, cross-linking, and redistribution of reactive functional groups. The magnitude and direction of these changes depend on the absorbed dose, the physical state of the material, its chemical composition, and the irradiation conditions. Previous work on humic acid solutions has demonstrated measurable and nonlinear radiochemical responses within the low-kilograys range. In particular, humic acid exposed to individual doses of 1, 2, 3, 4, and 5 kGy showed dose-dependent changes in chemiluminescence, absorption properties, and processes associated with molecular degradation or polymerization [16]. Other studies using higher irradiation ranges, including 10–100 kGy and 0–500 kGy, have confirmed progressive changes in molecular-weight behavior, carbon content, functional-group composition, and ion-complexation properties of humic substances [17]. These findings indicate that irradiation dose is a critical determinant of humic-material properties and provide a basis for examining whether comparatively mild irradiation can improve biological activity while avoiding the more extensive alteration associated with high absorbed doses.

Most previous research on irradiated humic substances has concentrated on physicochemical modification, molecular fractionation, contaminant complexation, or environmental applications [12–14]. Considerably less attention has been given to the use of gamma-irradiated humic acid as a crop biostimulant. In particular, evidence remains limited regarding whether irradiation-induced modification can simultaneously improve plant growth, photosynthetic performance, mineral nutrition, antioxidant status, yield formation, harvest timing, and fruit nutritional quality. Evaluation of these interconnected traits is necessary because improvement in a single physiological variable does not necessarily translate into higher marketable productivity or superior fruit quality.

A further limitation of conventional biostimulant studies is their frequent reliance on individual trait comparisons [18]. Plant responses to humic materials are inherently multidimensional, involving coordinated changes in root development, canopy growth, photosynthesis, nutrient acquisition, antioxidant metabolism, phenology, yield components, and fruit composition. Integrating univariate factorial analysis with correlation networks, hierarchical clustering, principal component analysis, and multi-criteria decision-making can provide a more rigorous assessment of treatment performance and identify whether a treatment produces balanced improvement across the whole plant system.

Accordingly, this study evaluated tomato responses to humic acid irradiated at 0, 2, or 5 kGy and applied at 2.5 or 5.0 kg ha^−1^, together with an untreated control. The objectives were to: determine the main and interactive effects of irradiation dose and humic acid application rate on vegetative growth, photosynthetic performance, leaf mineral composition, antioxidant-related traits, phenological development, marketable yield, and fruit quality; characterize relationships among the measured traits using multivariate analyses; and identify the most favorable treatment through an ensemble multi-criteria decision-making framework. We hypothesized that moderate gamma irradiation would enhance humic acid biostimulant activity, whereas a higher irradiation dose would attenuate the response, particularly at the greater application rate.

## Materials and methods

### Plant material, experimental conditions, and treatments

The experiment was conducted under greenhouse conditions at the University of Zanjan, Zanjan, Iran. Tomato (*Solanum lycopersicum* L.) cv. Super Strain B seedlings of uniform size and developmental stage were transplanted individually into 10-L plastic pots containing a sterilized mixture of loam soil, washed river sand, and peat moss at a ratio of 2:1:1 (v/v/v). Greenhouse conditions were maintained at 25 ± 2 °C during the day and 18 ± 2 °C at night, with 60–70% relative humidity and a 14-h photoperiod. Irrigation, basal mineral nutrition, pest management, and all other cultural practices were applied uniformly to all experimental units throughout the study. The experiment was arranged in a completely randomized design with seven treatments and three independent biological replicates per treatment. Each biological replicate consisted of three plants maintained as subsamples. Measurements obtained from the three plants were averaged to produce one experimental-unit value; therefore, the replicate mean, rather than the individual plant, was used in all statistical analyses. The six humic-acid-containing treatments formed a 3 × 2 factorial arrangement comprising three gamma irradiation doses applied to humic acid—0, 2, and 5 kGy—and two humic acid application rates—2.5 and 5.0 kg ha^−1^. An untreated treatment receiving no humic acid was included as an additional control (Table 1). Because irradiation dose is undefined in the absence of humic acid, the untreated control was not considered a zero-rate factorial combination and was excluded from the 3 × 2 factorial ANOVA.

**Table 1:** Experimental treatments and their roles in the statistical analysis.

Treatment	Irradiation dose	Humic acid rate
Untreated control	Not applicable	0 kg ha^−1^
Non-irradiated humic acid at 2.5 kg ha^−1^	0 kGy	2.5 kg ha^−1^
Non-irradiated humic acid at 5.0 kg ha^−1^	0 kGy	5.0 kg ha^−1^
Humic acid irradiated at 2 kGy and applied at 2.5 kg ha^−1^	2 kGy	2.5 kg ha^−1^
Humic acid irradiated at 2 kGy and applied at 5.0 kg ha^−1^	2 kGy	5.0 kg ha^−1^
Humic acid irradiated at 5 kGy and applied at 2.5 kg ha^−1^	5 kGy	2.5 kg ha^−1^
Humic acid irradiated at 5 kGy and applied at 5.0 kg ha^−1^	5 kGy	5.0 kg ha^−1^

### Humic acid source and gamma irradiation

The humic acid used in the experiment was a commercial agricultural-grade product supplied by Zist Nahadeh Pouya Company, Iran. The product was provided as a dark brown to black, water-soluble powder. According to the manufacturer’s certificate of analysis, it contained at least 70% humic acid and at least 85% organic matter on a dry-weight basis. The nominal treatment rates of 2.5 and 5.0 kg ha^−1^ were expressed on a commercial-product basis. Based on the minimum declared humic acid concentration, these rates corresponded to active humic acid equivalents of 1.75 and 3.50 kg ha^−1^, respectively.

Because the experiment was performed in pots, the field-equivalent rates were converted to the actual mass of commercial product supplied to each pot. The conversion was calculated from the effective substrate surface area of each pot using:


$$\begin{array}{c} {\text{M}}_{\text{pot}}={\text{R}}_{\text{field}}\times \text{100}\times {\text{A}}_{\text{pot}}, \end{array}$$

where M_pot_ is the commercial product mass applied per pot in mg, R_field_ is the nominal application rate in kg ha^−1^, 100 is the conversion factor from kg ha^−1^ to mg m^−2^, and A_pot_ is the internal surface area of the pot in m². For circular pots, surface area was calculated as:


$$\begin{array}{c} A_{pot}=\pi {\left(\frac{d}{\text{2}}\right)}^{\text{2}} \end{array}$$

The calculated amount of non-irradiated or gamma-irradiated humic acid product was dissolved or uniformly suspended in deionized water immediately before application. Each pot received a final treatment-solution volume of 200 mL at each application. The solution was applied uniformly to the substrate surface as a soil drench while avoiding direct contact with the stem. Control pots received the same volume of deionized water without humic acid. Gamma irradiation of the humic acid was performed at the Radiation Applications Research School, Nuclear Science and Technology Research Institute, Atomic Energy Organization of Iran, Tehran, Iran. Irradiation was conducted using a Gammacell 220 irradiator equipped with a cobalt-60 source (MDS Nordion, Ottawa, Canada). The humic acid was irradiated in the dry-powder state at nominal absorbed doses of 2 and 5 kGy. The dose rate at the sample position was 5.4 kGy h^−1^.

The nominal exposure duration was calculated using: t = D/˙D^,^ where t is the irradiation time, D is the target absorbed dose, and D˙ is the dose rate. Accordingly, the calculated exposure times were 0.370 h, equivalent to approximately 22.2 min, for the 2 kGy treatment and 0.926 h, equivalent to approximately 55.6 min, for the 5 kGy treatment. The non-irradiated humic acid was retained as the 0 kGy reference and was handled and stored under conditions comparable to those used for the irradiated materials. Following irradiation, the samples were stored in sealed, treatment-specific containers under dry and dark conditions at room temperature until preparation of the application suspensions. Dosimetry was performed using a Red 4034 Perspex dosimeter (Harwell Technologies, UK).

The first application was made seven days after transplanting, and three subsequent applications were performed at 14-day intervals. The rates of 2.5 and 5.0 kg ha^−1^ represented the total cumulative product amount applied during the experiment and were distributed equally among the four applications. Accordingly, one-quarter of the calculated pot-level dose was supplied at each application (S1 Table). Absorbed doses of 2 and 5 kGy were selected to represent two contrasting levels within a previously investigated low-kilograys range of gamma-induced humic acid modification. Goraczko and Sławiński [16] examined humic acid exposed to 1–5 kGy using a ^60^Co source and reported nonlinear dose-dependent changes in radiation-induced chemiluminescence and absorption characteristics, indicating that physicochemical modification was already detectable within this range. The 2 kGy treatment was therefore selected as a moderate irradiation level expected to induce comparatively limited radiolytic modification, whereas 5 kGy was selected as a stronger treatment at the upper boundary of that low-dose interval [16]. The selected doses were substantially below those used in several physicochemical studies of humic substances, in which irradiation levels of 10–100 kGy or 10–500 kGy caused marked changes in molecular-weight behavior, total organic carbon, functional-group distribution, and complexation properties [17]. The purpose of the present study was therefore not to determine the complete radiation dose–response curve of humic acid, but to compare two relatively mild irradiation levels and determine whether moderate modification improved its biostimulant effect on tomato. Non-irradiated humic acid was included as the 0-kGy reference.

### Characterization of raw and irradiated humic acid

The humic acid used in this study was a commercial agricultural grade product obtained from Zist Nahadeh Pouya Company. According to the supplier’s certificate of analysis, the material was a dark brown to black powder, with organic matter ≥85% (dry basis) and humic acid content ≥70% (dry basis). The product exhibited a cation exchange capacity of ~300 meq/100 g, indicating substantial capacity to interact with cations in solution. The typical elemental composition of similar humic acid products includes carbon as the major constituent along with oxygen, hydrogen, and minor nitrogen fractions, consistent with the heterogeneous molecular nature of humic substances. Humic acids are complex mixtures of molecules with a range of molecular weights and functional groups, including carboxyl and phenolic moieties that contribute to reactivity and bioactivity. After gamma irradiation at the selected doses (2 and 5 kGy), humic acid structural characteristics were expected to undergo partial radiolytic modification based on previous studies showing that irradiation induces changes in molecular‑weight distribution and functional group prevalence. Specifically, gamma irradiation has been shown to degrade larger humic molecules into smaller fractions and to alter the relative abundance of functional groups such as carboxyl and phenolic hydroxyl groups, with the degree of modification increasing with dose.

### Fourier-transform infrared spectroscopic characterization

Fourier-transform infrared spectroscopy was used to compare the functional-group profiles of non-irradiated humic acid and humic acid exposed to gamma-radiation doses of 2 and 5 kGy. Spectra were recorded over the wavenumber range of approximately 4000–400 cm^−1^ and expressed initially as percentage transmittance.

For comparative visualization, transmittance values were converted to absorbance according to:


$$\begin{array}{c} A=-{log}_{\text{1}\text{0}}\left(\frac{\%T}{\text{1}\text{0}\text{0}}\right) \end{array}$$

where A is absorbance and %T is percentage transmittance. Each spectrum was independently normalized to its maximum absorbance to facilitate comparison of band positions and spectral profiles among irradiation treatments. Because the spectra were normalized independently, differences in absolute band intensity were not interpreted quantitatively. Major absorption bands were identified from the instrument-generated spectra and assigned tentatively according to established functional-group regions of humic substances. The analysis focused on irradiation-associated shifts in band position, the appearance or disappearance of resolved bands, and changes in the overall spectral profile.

### Growing-substrate characterization and post-harvest soil analysis

Before pot filling, the loam soil, washed river sand, and peat moss were thoroughly homogenized at a ratio of 2:1:1 (v/v/v). A representative composite sample of the resulting growing substrate was obtained by combining ten subsamples collected from different positions within the homogenized substrate batch. The composite sample was air-dried at room temperature, passed through a 2-mm sieve, and analyzed to determine the initial physicochemical characteristics of the growing medium. Substrate pH was measured potentiometrically in a 1:2.5 substrate-to-deionized-water suspension after equilibration for 30 min. Electrical conductivity was determined in a 1:5 substrate-to-water extract using a conductivity meter. Organic matter was measured using the Walkley–Black wet-oxidation method. Total nitrogen was determined by the Kjeldahl method, whereas nitrate nitrogen was extracted with 2 M KCl and quantified colorimetrically. Available phosphorus was measured using sodium bicarbonate extraction according to the Olsen method, followed by molybdenum-blue colorimetric determination. Exchangeable potassium was extracted with 1 M ammonium acetate at pH 7.0 and quantified by flame photometry. Cation-exchange capacity was determined by ammonium acetate saturation and expressed as cmolc_cc kg^−1^. All nutrient concentrations were reported on an oven-dry substrate basis. The initial properties of the growing substrate are presented in S2 Table. At the end of the experiment, immediately after the final fruit harvest at 120 days after transplanting, post-harvest substrate samples were collected separately from each experimental unit. After removal of visible roots and plant residues, five subsamples were collected from different positions within each pot and combined to form one composite sample per biological replicate. The samples were air-dried, passed through a 2-mm sieve, and analyzed for pH, electrical conductivity, organic matter, total nitrogen, nitrate nitrogen, available phosphorus, exchangeable potassium, and cation-exchange capacity using the same procedures applied to the initial substrate. The initial substrate was represented by a single composite sample and was therefore used only as a descriptive baseline. It was not treated as an independently replicated experimental group or included in inferential statistical comparisons. Post-harvest substrate measurements from the three biological replicates were used as the observational units. The six humic-acid-containing treatments were analyzed as a 3 × 2 factorial arrangement comprising irradiation dose and humic acid application rate. The untreated control was excluded from the factorial model and compared with the factorial treatments using Dunnett-adjusted contrasts. A separate one-way treatment analysis followed by Tukey-adjusted comparisons was used when treatment-level groupings were required for tables or figures. Statistical significance was evaluated at P < 0.05.

### Growth and morphological measurements

Morphological observations were recorded at 30 days after transplanting (DAT) and, when repeated measurements were made, at 60 and 90 DAT. Plant height was measured from the substrate surface to the apical meristem. Stem diameter was measured with a digital caliper at 2 cm above the substrate surface. Leaf area was determined at 60 DAT using a portable leaf area meter on five fully expanded leaves per plant and expressed as cm² plant^−1^. At 120 DAT, the plants allocated to destructive sampling were removed from the pots; roots were washed free of substrate, blotted dry, oven-dried at 70 °C until constant mass, and expressed as g plant^−1^.

### Chlorophyll status and photosynthetic measurements

Chlorophyll index and gas-exchange measurements were obtained at 60 days after transplanting (DAT) from three recently fully expanded, healthy leaves per plant located in the upper-middle canopy. SPAD readings were collected using a SPAD-502 Plus chlorophyll meter, with three readings averaged per leaf. Net photosynthetic rate was measured between 09:00 and 11:00 using an LI-6400XT portable photosynthesis system at a photosynthetic photon flux density of 1000 µmol m^−2^ s^−1^, reference CO_2_ concentration of 400 µmol mol^−1^, leaf temperature of 25 °C, and relative humidity of 60–70%. The replicate mean was calculated from the three plant subsamples.

### Leaf nutrient and biochemical analyses

Leaf samples for mineral and biochemical analyses were collected at 60 days after transplanting (DAT) during the flowering and early fruit-setting stage between 09:00 and 11:00. For each biological replicate, nine recently fully expanded leaves from the upper-middle canopy were collected from the three subsample plants and pooled to form one replicate sample. The portion used for nitrogen, phosphorus, and potassium determination was washed, oven-dried at 70 °C to constant mass, ground, and analyzed using the Kjeldahl method for nitrogen, the molybdenum-blue colorimetric method with a UV–visible spectrophotometer for phosphorus, and flame photometry for potassium. A separate portion used for total soluble phenolics and DPPH antioxidant activity was immediately frozen in liquid nitrogen, stored at −80 °C, and extracted using 80% methanol at a tissue-to-solvent ratio of 1:10 (w/v) for 24 h at 4 °C in darkness. Total soluble phenolics were expressed as mg gallic acid equivalents g^−1^ DW, and DPPH radical-scavenging activity was expressed as percentage inhibition.

### Yield and harvest parameters

Days to first harvest were calculated from transplanting to the first harvest of marketable fruit. Fruits were harvested at the red-ripe stage, when at least 90% of the fruit surface had developed a uniform red color, at twice-weekly intervals from 67 days after transplanting (DAT) to 120 DAT. Fruit number and marketable fruit mass were recorded separately for each plant at every harvest and summed over the harvest period. The three plant values within each replicate were averaged before statistical analysis. Average fruit weight was calculated as cumulative marketable fruit mass divided by cumulative fruit number, and total marketable yield was expressed as kg plant^−1^. Yield increase relative to the untreated control was calculated only as a derived descriptive index and was not treated as an independent response variable in ANOVA.

### Fruit quality analyses

Fruit-quality analyses were performed at the third harvest, approximately 75 days after transplanting (DAT), using six uniformly red-ripe marketable fruits per biological replicate. Fruits were collected from the three subsample plants, combined to form one replicate sample, and analyzed immediately or stored at 4 °C for no longer than 24 h. Total soluble solids were measured using an Atago PAL-1 digital refractometer and expressed as °Brix. Titratable acidity was determined by titration with 0.1 N NaOH to pH 8.1 and expressed as percentage citric acid. Vitamin C was measured using the 2,6-dichlorophenolindophenol titrimetric method, and lycopene was quantified following extraction with a hexane:acetone:ethanol mixture (2:1:1, v/v/v) and spectrophotometric measurement at 503 nm; both were expressed as mg 100 g^−1^ FW.

### Statistical analysis

The six humic-acid-containing treatments were analyzed as a 3 × 2 factorial experiment, with gamma irradiation dose (0, 2, and 5 kGy), humic acid application rate (2.5 and 5.0 kg ha^−1^), and their interaction treated as fixed effects. For each trait, the mean of the three plant subsamples within each replicate was used as the observational unit (n = 4). The model was Y_ijk_ = μ + D_i_ + H_j_ + (D × H)_ij_ + ε_ijk_, where D_i_ is the effect of irradiation dose, H_j_ is the effect of humic acid rate, and (D × H)_ij_ is their interaction. Residual normality and homogeneity of variance were assessed using the Shapiro–Wilk and Levene tests, respectively, and data were transformed when necessary. Effect sizes, expressed as partial eta squared (partial η²), were reported together with F values, degrees of freedom, and exact P values.

When the irradiation dose × humic acid rate interaction was significant, the interaction was decomposed using simple-effect comparisons: humic acid rates were compared within each irradiation dose, and irradiation doses were compared within each humic acid rate. Tukey-adjusted pairwise comparisons were used at P < 0.05. The untreated no-humic-acid treatment was excluded from the factorial ANOVA and compared with each of the six factorial combinations using Dunnett-adjusted planned contrasts. Treatment means and dispersion statistics were calculated from the four biological replicates. Pearson correlation, hierarchical clustering, and principal component analysis were performed on replicate-derived treatment means as exploratory multivariate summaries. The mathematically derived variable ‘yield increase versus control’ was excluded from correlation and PCA analyses to avoid redundancy with total marketable yield.

Treatment means and standard deviations were calculated for all measured traits. Pearson correlation coefficients were computed to examine relationships among variables and were visualized using correlation heatmaps and network analyses. Hierarchical cluster analysis was performed using Z-score standardized data, average linkage clustering, and Euclidean distance. Principal component analysis (PCA) was conducted on standardized datasets to identify the major sources of variation among treatments.

To determine the best overall treatment, a multi-criteria decision-making framework comprising the Weighted Sum Model (WSM), Technique for Order Preference by Similarity to Ideal Solution (TOPSIS), VlseKriterijumska Optimizacija I Kompromisno Resenje (VIKOR), Multi-Objective Optimization on the Basis of Ratio Analysis (MOORA), and Complex Proportional Assessment (COPRAS) was applied. All analyses were conducted using Python-based scientific libraries, and statistical significance was assessed at P ≤ 0.05. All statistical and multivariate analyses were conducted using Python-based scientific libraries. Heatmaps, networks, dendrograms, PCA biplots, and MCDM ranking figures were created at a publication-quality resolution of 300 dpi.

## Results

### FTIR characterization of non-irradiated and gamma-irradiated humic acid

The FTIR spectra of non-irradiated humic acid and humic acid irradiated at 2 and 5 kGy retained the principal broad absorption regions characteristic of humic materials, but several dose-dependent shifts and changes in band resolution were observed (S3 Table). The non-irradiated humic acid showed major bands at 3515.66 and 3432.08 cm^−1^, a band at 1638.48 cm^−1^, and a low-wavenumber band at 587.82 cm^−1^. The broad region between approximately 3500 and 3400 cm^−1^ was tentatively assigned to hydrogen-bonded O–H stretching in phenolic, alcoholic, and carboxylic groups, with a possible contribution from N–H stretching. The band at approximately 1638 cm^−1^ may include overlapping contributions from aromatic C = C stretching, conjugated carbonyl groups, carboxylate structures, and absorbed water. Following irradiation at 2 kGy, the principal O–H-associated band occurred at 3440.48 cm^−1^, representing a small shift relative to the dominant non-irradiated band. Additional resolved bands appeared at 2923.79 and 2858.10 cm^−1^, which were tentatively assigned to asymmetric and symmetric stretching of aliphatic C–H groups, respectively. Further bands were detected at 1636.53, 1461.27, 1155.25, 1112.77, and 599.00 cm^−1^. The band at approximately 1461 cm^−1^ may reflect aliphatic C–H deformation and/or symmetric carboxylate stretching, whereas the bands between approximately 1155 and 1113 cm^−1^ are consistent with C–O and C–O–C vibrations in alcohol, phenolic, ether, and carbohydrate-like structures.

The spectrum of humic acid irradiated at 5 kGy showed bands at 3434.57, 1634.69, 1460.05, 1387.69, 1155.98, 1109.78, and 692.51 cm^−1^. The shift of the approximately 1638 cm^−1^ band to 1634.69 cm^−1^ and the appearance of a resolved band at 1387.69 cm^−1^ indicate modification of the chemical environments contributing to the aromatic, carbonyl, carboxylate, or aliphatic-deformation regions. The C–O-associated bands near 1156 and 1110 cm^−1^ remained detectable after irradiation, suggesting retention of oxygen-containing functional groups at both irradiation doses. Weak bands near 2166 and 2098 cm^−1^ occurred in the irradiated spectra. Because these bands are not highly diagnostic of the principal humic-acid functional groups and may represent combination bands, minor components, or instrumental effects, they were not assigned to a specific irradiation-induced chemical structure. The persistence of the broad O–H region, the approximately 1635–1638 cm^−1^ band, and the C–O-associated fingerprint bands indicated that the major functional framework of the humic material was retained after irradiation. However, the small shifts in band position and changes in band resolution demonstrated that gamma irradiation modified the local chemical environments of some functional groups. These spectral changes were dose dependent, but the FTIR data alone could not establish molecular fragmentation, specific bond cleavage, or molecular-weight reduction.

### Effects of irradiation dose, humic acid rate, and their interaction

**Fig 1:**
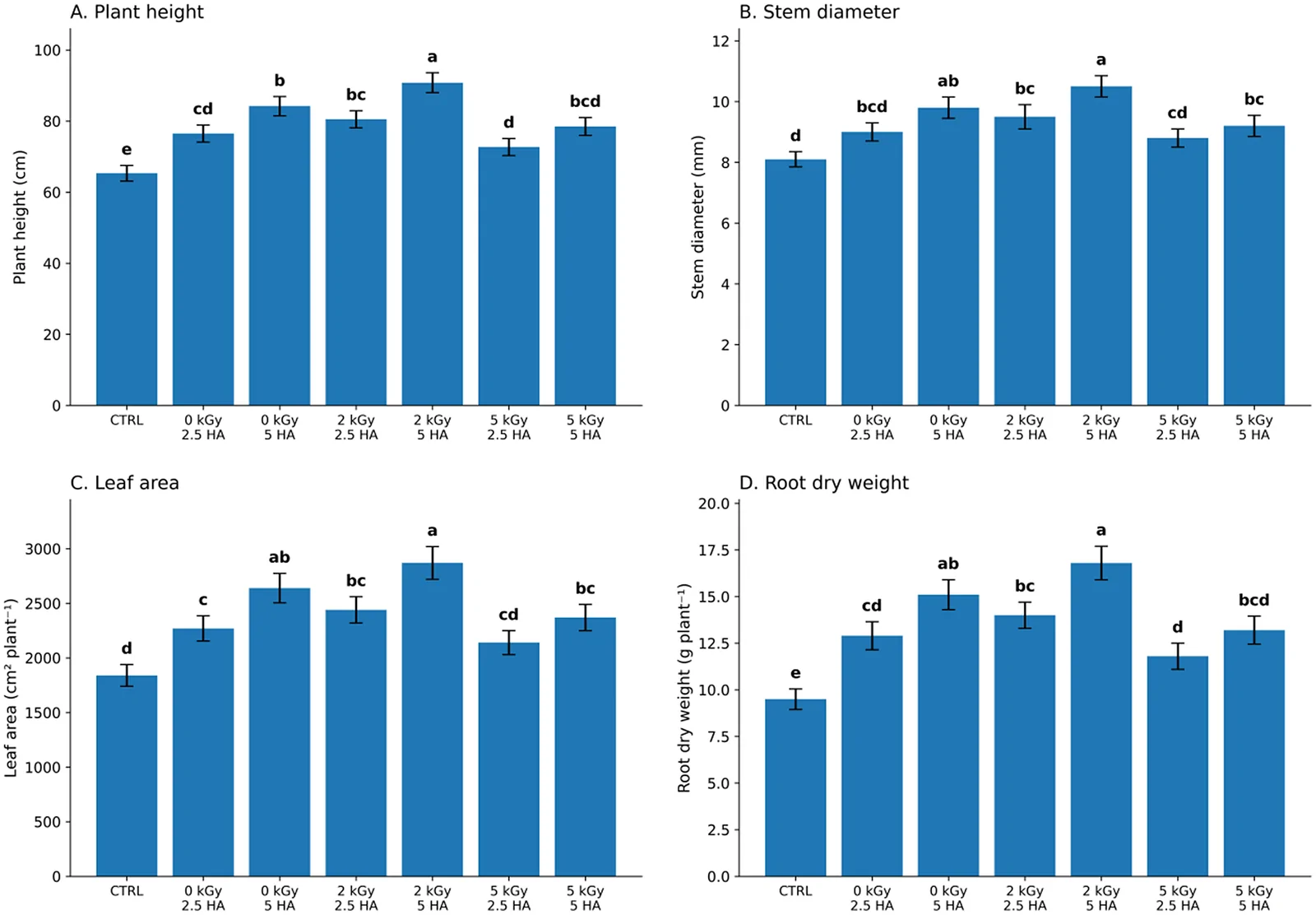
Effects of gamma irradiation dose and humic acid application rate on vegetative growth of tomato plants. (A) Plant height, (B) stem diameter, (C) leaf area, and (D) root dry weight of tomato plants treated with non-irradiated humic acid or humic acid irradiated at 2 or 5 kGy and applied at 2.5 or 5.0 kg ha^−1^. Bars represent means ± standard deviation (SD; n = 3). Different lowercase letters above the bars indicate significant differences among treatments according to Tukey’s honestly significant difference test at P < 0.05. CTRL represents the untreated control. Treatment labels: CTRL, untreated control; 0 kGy–2.5 HA, non-irradiated humic acid applied at 2.5 kg ha^−1^; 0 kGy–5 HA, non-irradiated humic acid applied at 5.0 kg ha^−1^; 2 kGy–2.5 HA, humic acid irradiated at 2 kGy and applied at 2.5 kg ha^−1^; 2 kGy–5 HA, humic acid irradiated at 2 kGy and applied at 5.0 kg ha^−1^; 5 kGy–2.5 HA, humic acid irradiated at 5 kGy and applied at 2.5 kg ha^−1^; and 5 kGy–5 HA, humic acid irradiated at 5 kGy and applied at 5.0 kg ha^−1^.

**Fig 2:**
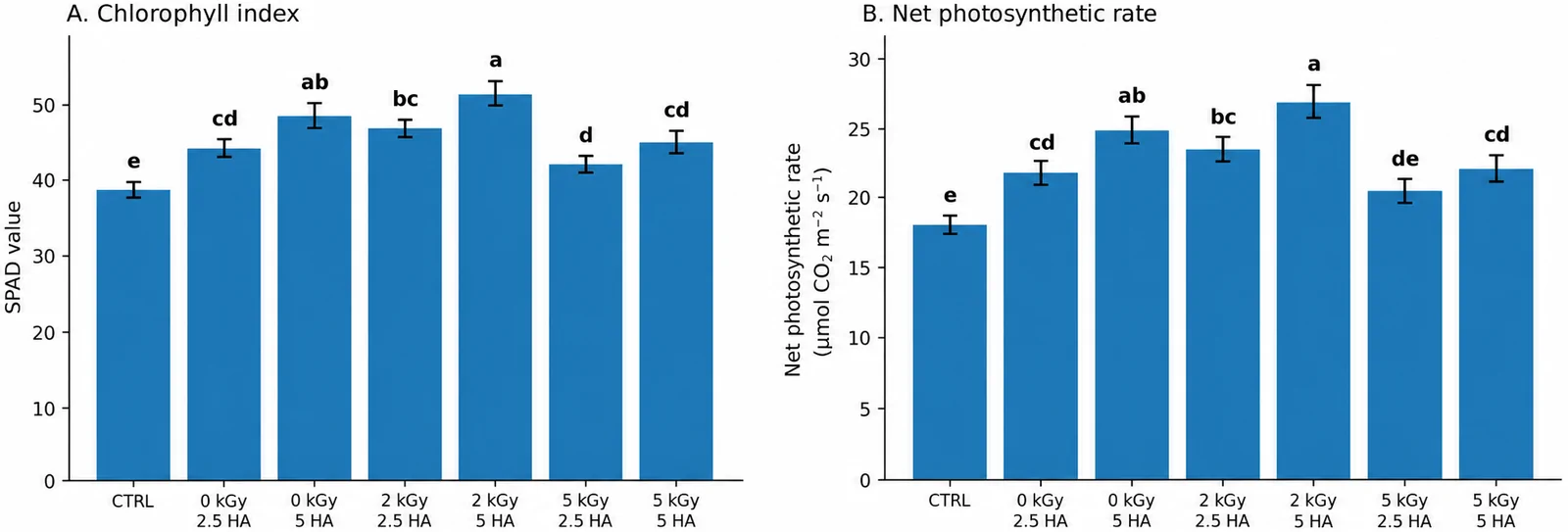
Effects of gamma irradiation dose and humic acid application rate on chlorophyll status and photosynthetic performance of tomato plants. (A) Chlorophyll index and (B) net photosynthetic rate of tomato plants treated with non-irradiated humic acid or humic acid irradiated at 2 or 5 kGy and applied at 2.5 or 5.0 kg ha^−1^. Bars represent means ± standard deviation (SD; n = 3). Different lowercase letters above the bars indicate significant differences among treatments according to Tukey’s honestly significant difference test at P < 0.05. CTRL represents the untreated control.

**Fig 3:**
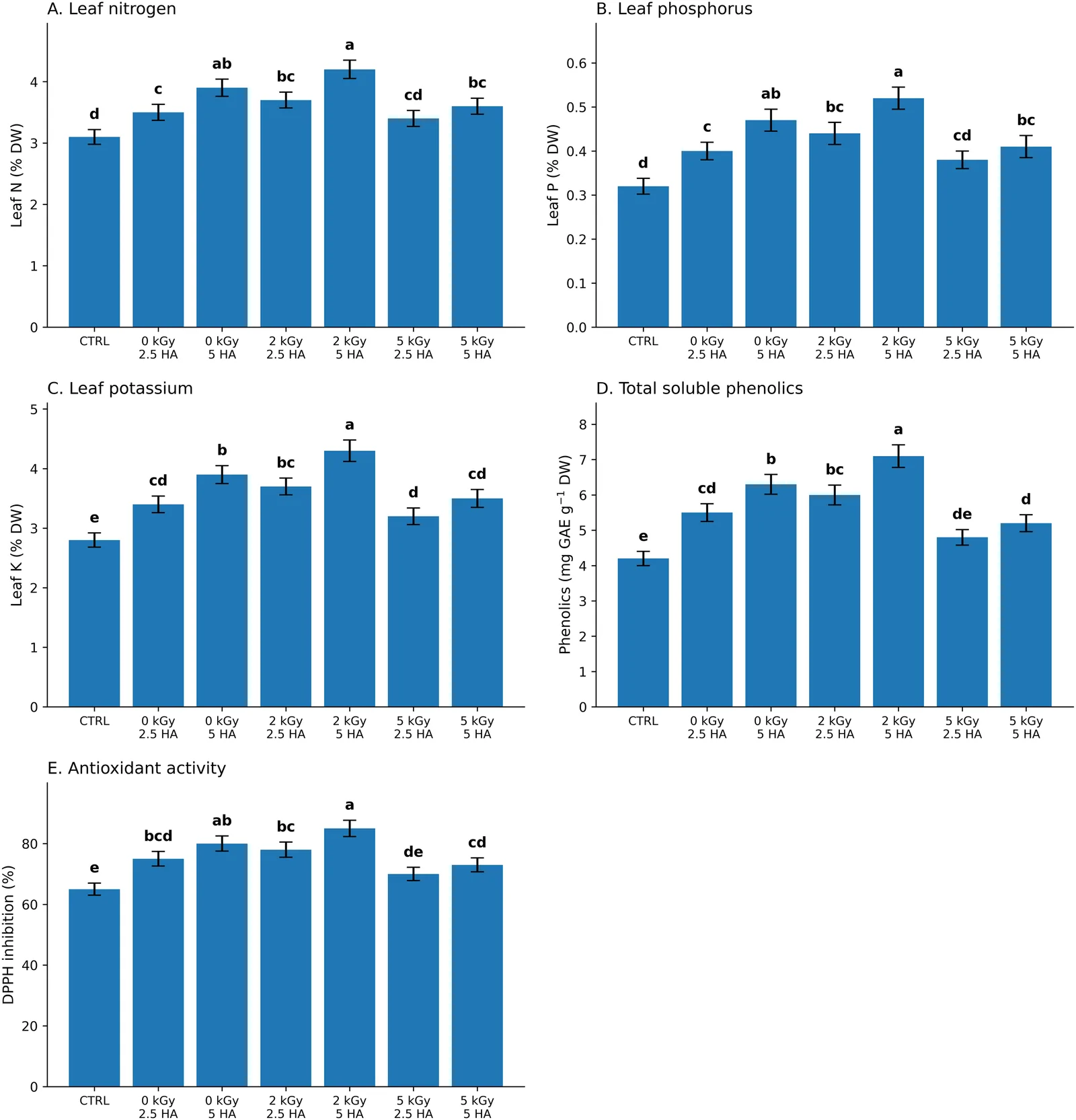
Effects of gamma irradiation dose and humic acid application rate on leaf nutrient status and antioxidant-related traits of tomato plants. (A) Leaf nitrogen concentration, (B) leaf phosphorus concentration, (C) leaf potassium concentration, (D) total soluble phenolic content, and (E) DPPH radical-scavenging activity of tomato plants treated with non-irradiated humic acid or humic acid irradiated at 2 or 5 kGy and applied at 2.5 or 5.0 kg ha^−1^. Bars represent means ± standard deviation (SD; n = 3). Different lowercase letters above the bars indicate significant differences among treatments according to Tukey’s honestly significant difference test at P < 0.05. CTRL represents the untreated control.

**Fig 4:**
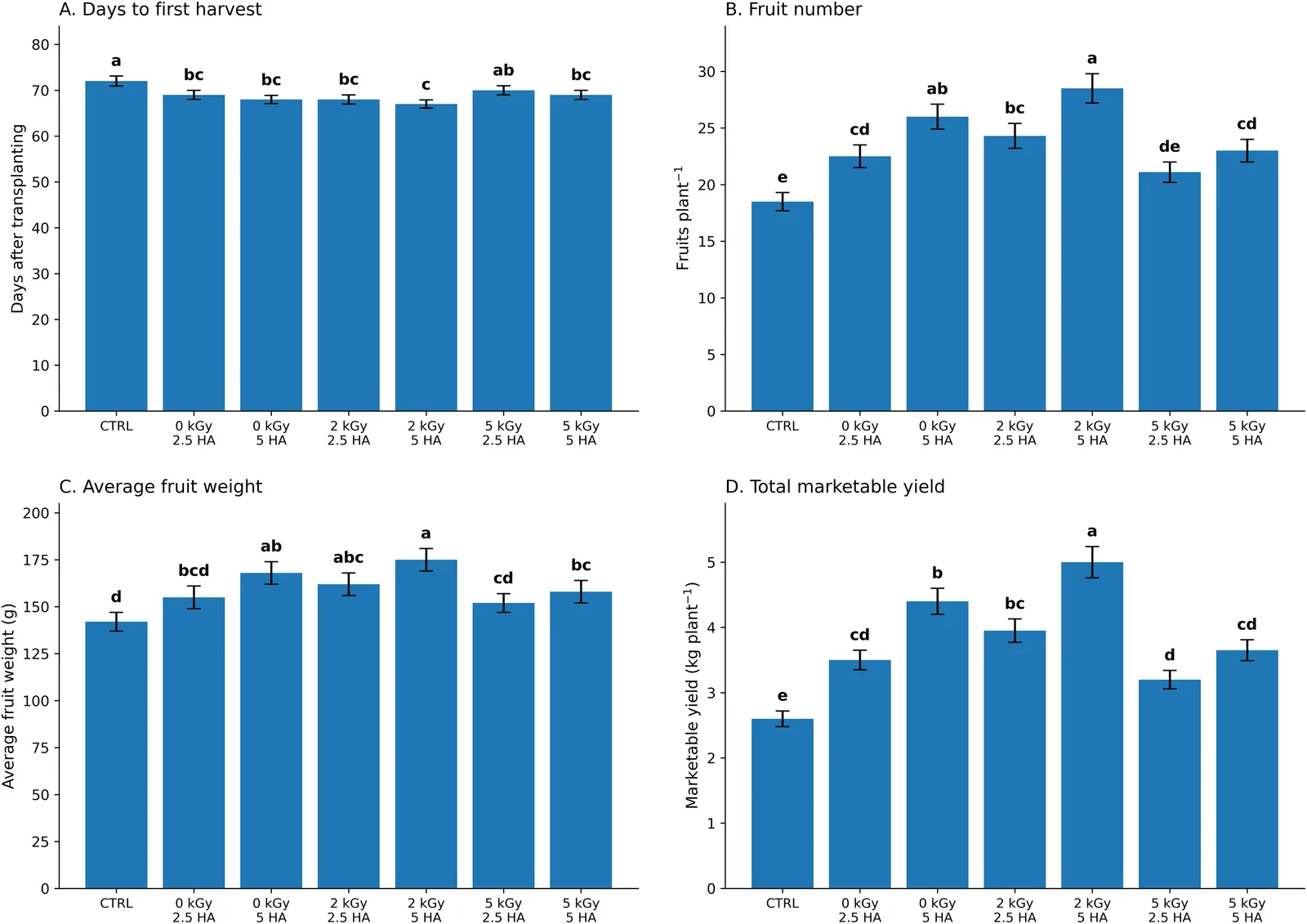
Effects of gamma irradiation dose and humic acid application rate on phenological development, yield components, and marketable yield of tomato plants. (A) Days to first harvest, (B) number of fruits per plant, (C) average fruit weight, and (D) total marketable yield of tomato plants treated with non-irradiated humic acid or humic acid irradiated at 2 or 5 kGy and applied at 2.5 or 5.0 kg ha^−1^. Bars represent means ± standard deviation (SD; n = 3). Different lowercase letters above the bars indicate significant differences among treatments according to Tukey’s honestly significant difference test at P < 0.05. CTRL represents the untreated control. The significant gamma irradiation dose × humic acid application rate interaction detected for total marketable yield is presented in panel D.

**Fig 5:**
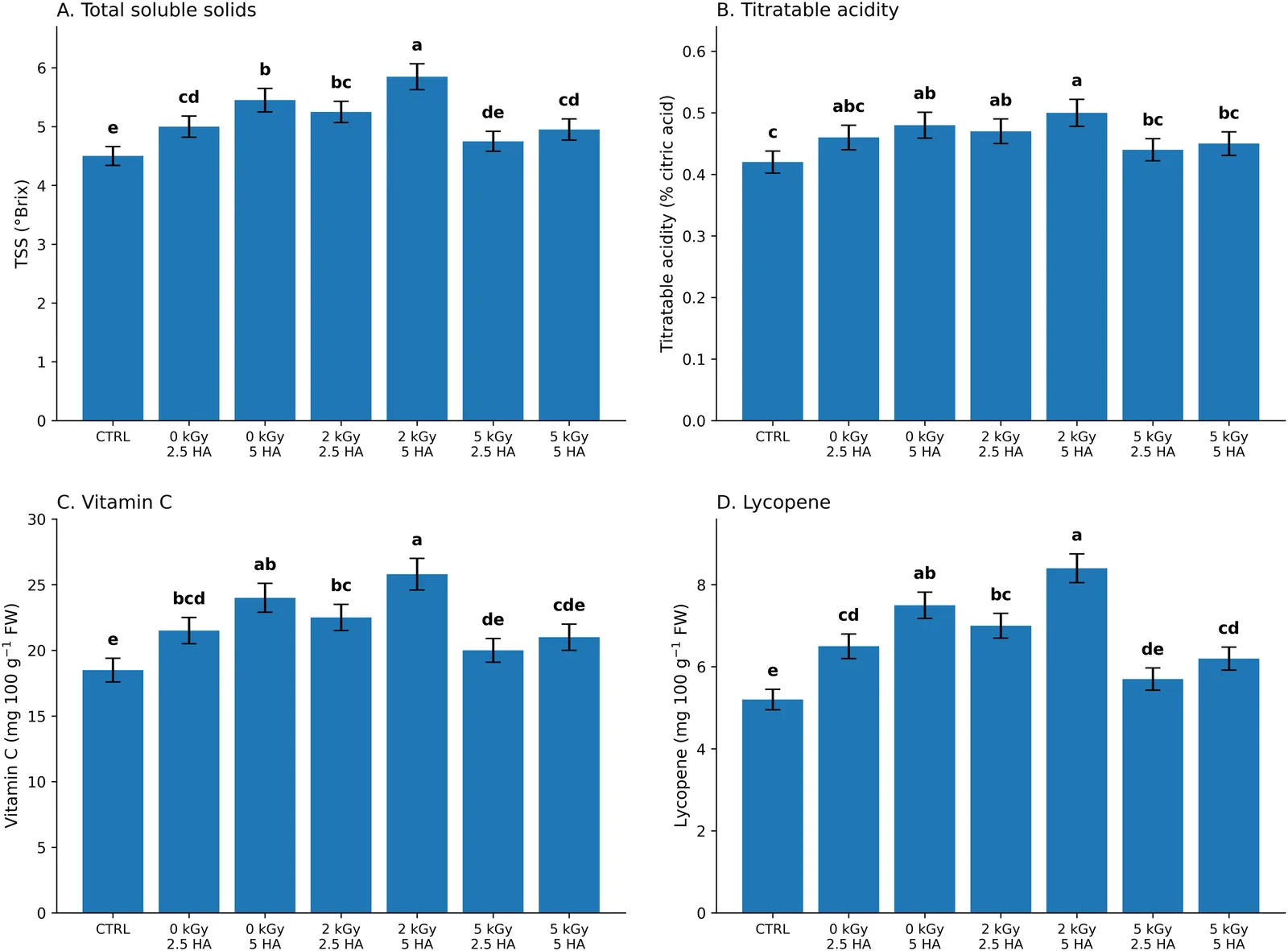
Effects of gamma irradiation dose and humic acid application rate on tomato fruit-quality attributes. (A) Total soluble solids, (B) titratable acidity, (C) vitamin C concentration, and (D) lycopene concentration in fruits produced by plants treated with non-irradiated humic acid or humic acid irradiated at 2 or 5 kGy and applied at 2.5 or 5.0 kg ha^−1^. Bars represent means ± standard deviation (SD; n = 3). Different lowercase letters above the bars indicate significant differences among treatments according to Tukey’s honestly significant difference test at P < 0.05. CTRL represents the untreated control.

The six humic-acid-containing treatments were analyzed as a 3 × 2 factorial arrangement comprising three gamma-irradiation doses and two humic acid application rates. The untreated control was excluded from the factorial analysis but retained in the treatment-level Tukey and Dunnett comparisons. Treatment means are presented as mean ± SD, and different lowercase letters in Figs 1–5 indicate significant differences according to Tukey’s honestly significant difference test at P < 0.05. Gamma-irradiation dose and humic acid application rate significantly affected most growth, physiological, nutritional, biochemical, yield, and fruit-quality traits. The irradiation dose × humic acid rate interaction was significant only for total marketable yield. No significant interaction was detected for the remaining traits, indicating that their responses to humic acid rate were statistically consistent across irradiation doses within the precision of the present dataset.

### Effects of gamma-irradiated humic acid on tomato vegetative growth

Gamma irradiation dose and humic acid application rate significantly affected plant height, stem diameter, leaf area, and root dry weight, whereas their interaction was not significant for any of these variables, with interaction P values ranging from 0.278 to 0.385 (Fig 1). Humic acid irradiated at 2 kGy and applied at 5.0 kg ha^−1^ produced the greatest vegetative growth, with a plant height of 90.7 ± 2.72 cm, stem diameter of 10.5 ± 0.36 mm, leaf area of 2880 ± 143.82 cm² plant^−1^, and root dry weight of 16.8 ± 0.92 g plant^−1^. This treatment was assigned to the highest Tukey group for all four traits. Non-irradiated humic acid applied at 5.0 kg ha^−1^ also produced comparatively high stem diameter, leaf area, and root dry weight and was statistically comparable with humic acid irradiated at 2 kGy and applied at 5.0 kg ha^−1^ for these traits. However, its plant height was significantly lower. The untreated control produced the lowest plant height, leaf area, and root dry weight, with respective means of 65.2 ± 2.01 cm, 1850 ± 92.33 cm² plant^−1^, and 9.5 ± 0.53 g plant^−1^. Among the humic-acid-containing treatments, humic acid irradiated at 5 kGy and applied at 2.5 kg ha^−1^ produced the weakest vegetative response. Its stem diameter, at 8.8 ± 0.26 mm, did not differ significantly from that of the untreated control.

### Photosynthetic performance and chlorophyll responses

Both gamma irradiation dose and humic acid application rate significantly affected chlorophyll index and net photosynthetic rate, whereas their interaction was not significant for either chlorophyll index (P = 0.398) or net photosynthetic rate (P = 0.230; Fig 2). Humic acid irradiated at 2 kGy and applied at 5.0 kg ha^−1^ produced the highest chlorophyll index and net photosynthetic rate, reaching 51.3 ± 1.32 SPAD units and 26.8 ± 1.06 µmol CO_2_ m^−2^ s^−1^, respectively. Non-irradiated humic acid applied at 5.0 kg ha^−1^ produced 48.5 ± 1.25 SPAD units and a net photosynthetic rate of 24.9 ± 0.98 µmol CO_2_ m^−2^ s^−1^. These values were statistically comparable with those obtained from humic acid irradiated at 2 kGy and applied at 5.0 kg ha^−1^, as indicated by their overlapping Tukey groups. The untreated control had the lowest chlorophyll index and net photosynthetic rate, at 38.5 ± 0.96 SPAD units and 18.1 ± 0.72 µmol CO_2_ m^−2^ s^−1^, respectively. All six humic-acid-containing treatments significantly increased both variables relative to the untreated control in the Dunnett-adjusted comparisons. Among the irradiated treatments, humic acid irradiated at 5 kGy and applied at 2.5 kg ha^−1^ produced the lowest chlorophyll index and photosynthetic rate, demonstrating that the higher irradiation dose was less effective when combined with the lower humic acid application rate.

### Leaf nutrient status and antioxidant responses

Leaf nitrogen, phosphorus, and potassium concentrations were significantly affected by gamma irradiation dose and humic acid application rate. Total soluble phenolic content and DPPH radical-scavenging activity were similarly affected by both factors. However, no significant gamma irradiation dose × humic acid application rate interaction was detected for leaf nitrogen, phosphorus, potassium, total soluble phenolics, or antioxidant activity, with interaction P values ranging from 0.111 to 0.365 (Fig 3). Humic acid irradiated at 2 kGy and applied at 5.0 kg ha^−1^ produced the highest leaf nitrogen, phosphorus, and potassium concentrations, reaching 4.20 ± 0.15%, 0.52 ± 0.03%, and 4.30 ± 0.17%, respectively. The same treatment also produced the highest total soluble phenolic content, at 7.10 ± 0.32 mg GAE g^−1^ DW, and the highest DPPH radical-scavenging activity, at 85.0 ± 2.57%. Non-irradiated humic acid applied at 5.0 kg ha^−1^ also produced comparatively high nutrient concentrations and antioxidant responses. Its leaf nitrogen, phosphorus, and DPPH activity were statistically comparable with those obtained from humic acid irradiated at 2 kGy and applied at 5.0 kg ha^−1^, whereas its potassium and total soluble phenolic contents were significantly lower. Humic acid irradiated at 2 kGy and applied at 2.5 kg ha^−1^ generally occupied an intermediate statistical group between the highest-performing treatment and the less effective treatments. The untreated control produced the lowest nutrient and biochemical values, including 3.10 ± 0.11% nitrogen, 0.32 ± 0.02% phosphorus, 2.80 ± 0.11% potassium, 4.20 ± 0.19 mg GAE g^−1^ DW total soluble phenolics, and 65.0 ± 1.97% DPPH inhibition. Humic acid irradiated at 5 kGy and applied at 2.5 kg ha^−1^ produced the weakest biochemical response among the humic-acid-containing treatments. It did not differ significantly from the untreated control for leaf nitrogen, total soluble phenolic content, or DPPH activity, although its leaf phosphorus and potassium concentrations remained significantly greater than those of the untreated control.

### Effects on phenology and yield components

Days to first harvest were significantly affected by gamma irradiation dose (F_2,12_ = 6.00, P = 0.0156), whereas the main effect of humic acid application rate was not significant at the 5% level (P = 0.0554). The gamma irradiation dose × humic acid application rate interaction was also not significant (P = 1.000; Fig 4A). The untreated control required the longest period to reach the first marketable harvest, at 72 ± 1 days after transplanting. Humic acid irradiated at 2 kGy and applied at 5.0 kg ha^−1^ resulted in the earliest harvest, at 67 ± 1 days, and was assigned to a significantly earlier Tukey group than the untreated control. Non-irradiated humic acid applied at 5.0 kg ha^−1^ and humic acid irradiated at 2 kGy and applied at 2.5 kg ha^−1^ reached the first harvest at 68 ± 1 days. Non-irradiated humic acid applied at 2.5 kg ha^−1^ and humic acid irradiated at 5 kGy and applied at 5.0 kg ha^−1^ reached harvest at 69 ± 1 days. Humic acid irradiated at 5 kGy and applied at 2.5 kg ha^−1^ required 70 ± 1 days and did not differ significantly from the untreated control in the Dunnett-adjusted comparison. Gamma irradiation dose and humic acid application rate significantly affected fruit number and average fruit weight, whereas their interaction was not significant for fruit number (P = 0.194) or average fruit weight (P = 0.530). Humic acid irradiated at 2 kGy and applied at 5.0 kg ha^−1^ produced the greatest number of fruits, at 28.5 ± 1.35 fruits plant^−1^, followed by non-irradiated humic acid applied at 5.0 kg ha^−1^, at 26.1 ± 1.15 fruits plant^−1^. These two treatments were statistically comparable. The untreated control produced the fewest fruits, at 18.5 ± 0.82 fruits plant^−1^. Average fruit weight was also highest under humic acid irradiated at 2 kGy and applied at 5.0 kg ha^−1^, reaching 175 ± 6.13 g. This value was statistically comparable with those obtained from non-irradiated humic acid applied at 5.0 kg ha^−1^ and humic acid irradiated at 2 kGy and applied at 2.5 kg ha^−1^, which produced average fruit weights of 168 ± 5.88 and 162 ± 5.68 g, respectively. The untreated control produced the lowest average fruit weight, at 142 ± 4.95 g. Humic acid irradiated at 5 kGy and applied at 2.5 kg ha^−1^ produced an average fruit weight of 152 ± 5.31 g and did not differ significantly from the untreated control according to the Dunnett-adjusted comparison.

### Interaction effect on total marketable yield

Total marketable yield was significantly affected by gamma irradiation dose (F_2,12_ = 50.10, P < 0.001, partial η² = 0.893), humic acid application rate (F_1,12_ = 83.82, P < 0.001, partial η² = 0.875), and their interaction (F_2,12_ = 5.05, P = 0.0257, partial η² = 0.457). The significant interaction demonstrated that the effect of increasing humic acid application rate depended on the irradiation dose applied to the humic acid. For non-irradiated humic acid, increasing the application rate from 2.5 to 5.0 kg ha^−1^ increased total marketable yield from 3.51 ± 0.16 to 4.38 ± 0.20 kg plant^−1^. This difference was significant after Tukey adjustment (P = 0.0008). For humic acid irradiated at 2 kGy, increasing the application rate from 2.5 to 5.0 kg ha^−1^ increased yield from 3.94 ± 0.18 to 4.99 ± 0.23 kg plant^−1^, and this difference was also significant (P = 0.0001). In contrast, for humic acid irradiated at 5 kGy, increasing the application rate from 2.5 to 5.0 kg ha^−1^ increased yield from 3.22 ± 0.14 to 3.63 ± 0.16 kg plant^−1^, but this difference was not significant (P = 0.1268). At the 5.0 kg ha^−1^ humic acid application rate, humic acid irradiated at 2 kGy produced significantly greater yield than both non-irradiated humic acid (P = 0.0131) and humic acid irradiated at 5 kGy (P < 0.0001). Non-irradiated humic acid applied at 5.0 kg ha^−1^ also produced significantly greater yield than humic acid irradiated at 5 kGy and applied at the same rate (P = 0.0027). At the 2.5 kg ha^−1^ humic acid application rate, humic acid irradiated at 2 kGy produced significantly greater yield than humic acid irradiated at 5 kGy (P = 0.0038), whereas its difference from non-irradiated humic acid was not significant. Across all seven treatments, humic acid irradiated at 2 kGy and applied at 5.0 kg ha^−1^ produced the highest total marketable yield and was assigned exclusively to Tukey group “a.” Non-irradiated humic acid applied at 5.0 kg ha^−1^ ranked second, at 4.38 ± 0.20 kg plant^−1^, whereas the untreated control produced the lowest yield, at 2.63 ± 0.12 kg plant^−1^. All six humic-acid-containing treatments produced significantly greater total marketable yield than the untreated control in the Dunnett-adjusted comparisons.

### Fruit quality attributes

Gamma irradiation dose and humic acid application rate significantly affected total soluble solids, titratable acidity, vitamin C, and lycopene content (Fig 5). No significant gamma irradiation dose × humic acid application rate interaction was detected for total soluble solids (P = 0.0659), titratable acidity (P = 0.579), vitamin C (P = 0.202), or lycopene (P = 0.0743). Accordingly, treatment differences in these traits were predominantly associated with the independent effects of irradiation dose and humic acid application rate rather than a statistically confirmed interaction. Humic acid irradiated at 2 kGy and applied at 5.0 kg ha^−1^ produced the highest total soluble solids, at 5.90 ± 0.15 °Brix, and was significantly superior to the remaining treatments. This treatment also produced the highest titratable acidity, vitamin C, and lycopene concentrations, reaching 0.50 ± 0.02% citric acid, 25.8 ± 1.05 mg 100 g^−1^ FW, and 8.30 ± 0.37 mg 100 g^−1^ FW, respectively. For titratable acidity, non-irradiated humic acid applied at 5.0 kg ha^−1^ and humic acid irradiated at 2 kGy and applied at 2.5 kg ha^−1^ were statistically comparable with humic acid irradiated at 2 kGy and applied at 5.0 kg ha^−1^. Non-irradiated humic acid applied at 5.0 kg ha^−1^ was also statistically comparable with humic acid irradiated at 2 kGy and applied at 5.0 kg ha^−1^ for vitamin C and lycopene, producing 24.0 ± 0.96 mg 100 g^−1^ FW vitamin C and 7.50 ± 0.34 mg 100 g^−1^ FW lycopene. However, its total soluble solids content of 5.50 ± 0.13 °Brix was significantly lower. The untreated control produced the lowest total soluble solids, vitamin C, and lycopene values, at 4.50 ± 0.11 °Brix, 18.5 ± 0.78 mg 100 g^−1^ FW, and 5.20 ± 0.24 mg 100 g^−1^ FW, respectively. Humic acid irradiated at 5 kGy and applied at 2.5 kg ha^−1^ consistently produced the weakest fruit-quality response among the humic-acid-containing treatments and did not differ significantly from the untreated control for total soluble solids, titratable acidity, vitamin C, or lycopene. Overall, humic acid irradiated at 2 kGy and applied at 5.0 kg ha^−1^ produced the most favorable combined response across the measured fruit-quality attributes.

### Trait correlation network analysis

**Fig 6:**
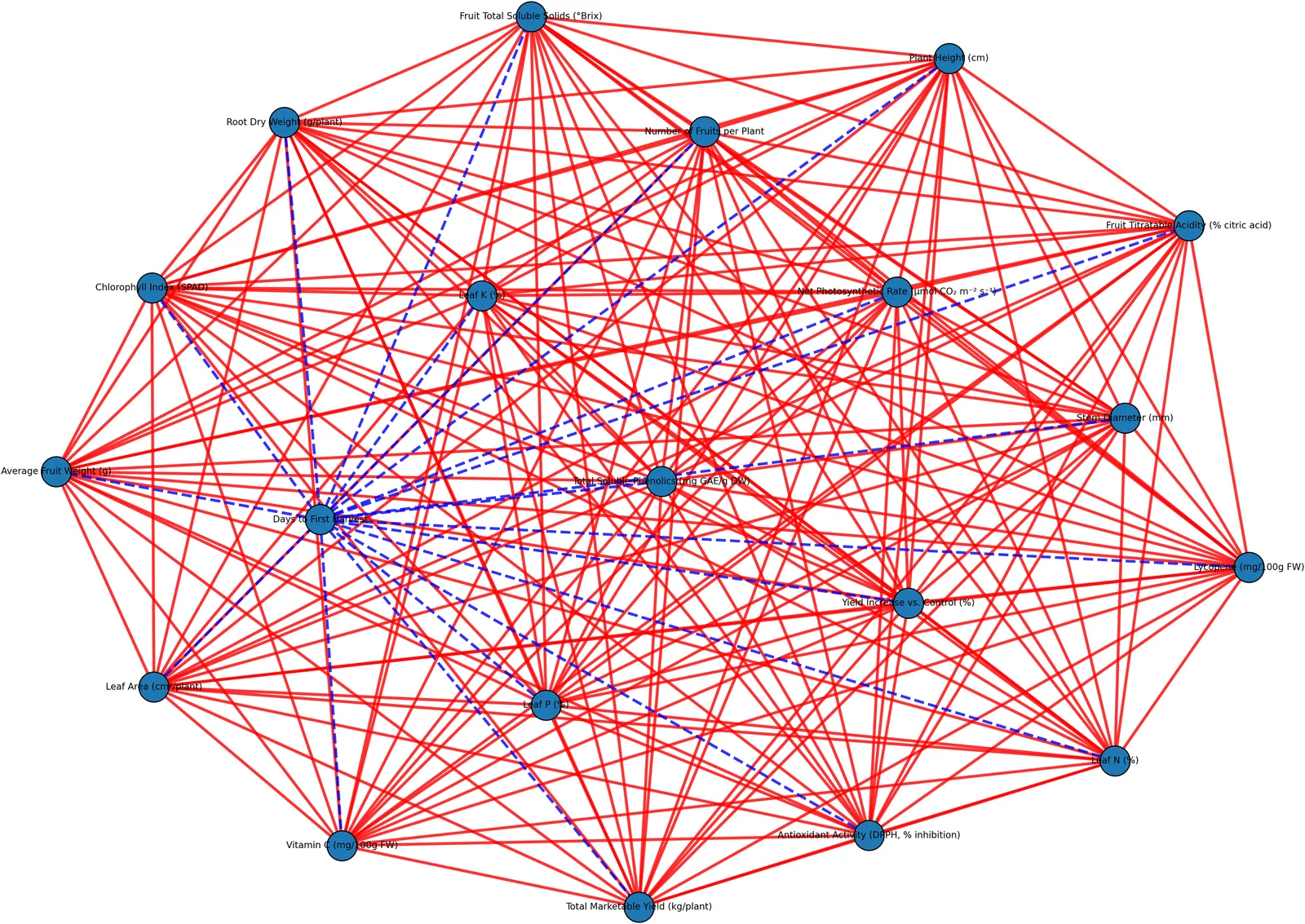
Signed trait correlation network illustrating positive and negative associations among growth, physiological, nutritional, yield, and fruit quality traits of tomato under humic acid and gamma irradiation treatments. Red solid edges represent positive correlations and blue dashed edges represent negative correlations (|r| ≥ 0.75). Edge thickness is proportional to correlation strength.

To identify coordinated relationships among tomato growth, physiological, nutritional, biochemical, phenological, yield, and fruit-quality traits, Pearson correlations were calculated after centering each trait within treatment. This treatment adjustment removed the common shifts generated by differences among the seven treatment means and allowed the network to represent covariation among replicate deviations rather than merely reproducing the overall treatment-performance gradient. The derived variable “yield increase relative to control” was excluded because it was mathematically dependent on total marketable yield. The network retained relationships with |r| ≥ 0.60 and P < 0.05. The treatment-adjusted network contained 19 traits connected by 25 strong relationships, comprising 18 positive and seven negative edges (Fig 6). The resulting structure linked vegetative growth and photosynthetic performance with fruit production and marketable yield, while days to first harvest formed a contrasting phenological component characterized exclusively by negative connections. Stem diameter and leaf area exhibited the strongest positive relationship in the network (r = 0.912, P < 0.001), demonstrating close coordination between radial stem growth and canopy expansion. Leaf area was also positively related to chlorophyll index (r = 0.715, P = 0.004), leaf potassium concentration (r = 0.709, P = 0.005), and total marketable yield (r = 0.697, P = 0.006). These associations indicate that replicate-level increases in canopy development were accompanied by improved chlorophyll status, potassium accumulation, and fruit productivity. Plant height was similarly positively associated with leaf potassium concentration (r = 0.647, P = 0.013), fruit number (r = 0.622, P = 0.018), total marketable yield (r = 0.683, P = 0.007), and vitamin C concentration (r = 0.604, P = 0.022). Photosynthetic performance was closely connected to reproductive output. Net photosynthetic rate showed a strong positive relationship with fruit number per plant (r = 0.887, P < 0.001), representing the second-strongest association in the network. Net photosynthetic rate was also positively associated with total marketable yield (r = 0.776, P = 0.001) and fruit lycopene concentration (r = 0.628, P = 0.016). These relationships indicate that plants maintaining greater carbon-assimilation capacity also tended to produce more fruits, greater marketable yield, and fruits with higher lycopene accumulation. Chlorophyll index was positively related to both leaf area and stem diameter, with the latter association reaching r = 0.681 (P = 0.007). Total marketable yield was one of the principal network hubs, with six retained connections. Yield was positively related to net photosynthetic rate, leaf area, plant height, fruit number, and average fruit weight. The positive relationships of total marketable yield with fruit number (r = 0.695, P = 0.006) and average fruit weight (r = 0.683, P = 0.007) demonstrate that both reproductive components contributed to productivity variation. Root dry weight was also positively associated with fruit number (r = 0.612, P = 0.020), suggesting that stronger belowground development supported greater reproductive output. Fruit number represented another major hub, with six network connections. In addition to its positive associations with net photosynthetic rate, total marketable yield, plant height, and root dry weight, fruit number was positively related to lycopene concentration (r = 0.643, P = 0.013). This relationship indicates that increased fruit production was not accompanied by reduced lycopene concentration within the range represented by the treatments. Lycopene was also positively associated with net photosynthetic rate, linking photosynthetic carbon acquisition to fruit pigment accumulation. The nutrient-related traits formed more selective connections after treatment adjustment. Leaf phosphorus and potassium concentrations were positively related (r = 0.621, P = 0.018), indicating coordinated accumulation of these macronutrients. Leaf potassium was additionally associated with plant height and leaf area, supporting its close relationship with vegetative expansion. Leaf nitrogen did not retain any relationship at the selected |r| ≥ 0.60 threshold after removal of treatment-level effects. This does not indicate an absence of biological association but shows that its residual correlations were weaker than the predefined network criterion. Days to first harvest was the most highly connected trait in the network, with seven exclusively negative relationships. Earlier harvest, represented by lower values for days to first harvest, was associated with greater leaf area (r = −0.785, P < 0.001), total marketable yield (r = −0.749, P = 0.002), fruit number (r = −0.696, P = 0.006), plant height (r = −0.679, P = 0.008), net photosynthetic rate (r = −0.645, P = 0.013), stem diameter (r = −0.641, P = 0.013), and root dry weight (r = −0.621, P = 0.018). Consequently, plants exhibiting stronger vegetative growth, enhanced photosynthetic performance, and greater reproductive output generally reached marketable harvest earlier. Network-centrality analysis identified days to first harvest as the most connected node, with a degree of seven and a weighted strength of 4.817. Total marketable yield and fruit number each had six connections, with weighted strengths of 4.283 and 4.156, respectively. Leaf area and plant height each had five retained associations, followed by net photosynthetic rate and stem diameter with four connections each. These traits therefore represented the principal structural hubs linking vegetative development, physiological performance, phenological timing, and fruit productivity.

The coordinated treatment responses were further supported by the treatment-adjusted correlation network. Net photosynthetic rate was strongly and positively associated with fruit number per plant (r = 0.887, P < 0.001) and total marketable yield (r = 0.776, P = 0.001), indicating that replicate-level variation in carbon assimilation was closely aligned with reproductive output. Net photosynthetic rate was also positively associated with lycopene concentration (r = 0.628, P = 0.016), linking leaf physiological performance with fruit nutritional quality. Total marketable yield was positively associated with both fruit number (r = 0.695, P = 0.006) and average fruit weight (r = 0.683, P = 0.007), demonstrating that treatment-dependent yield variation reflected simultaneous changes in fruit production and fruit size. Leaf area was positively associated with marketable yield (r = 0.697, P = 0.006), whereas days to first harvest was negatively associated with net photosynthetic rate (r = −0.645, P = 0.013), fruit number (r = −0.696, P = 0.006), and marketable yield (r = −0.749, P = 0.002).

### Cluster-based comparison of treatments

**Fig 7:**
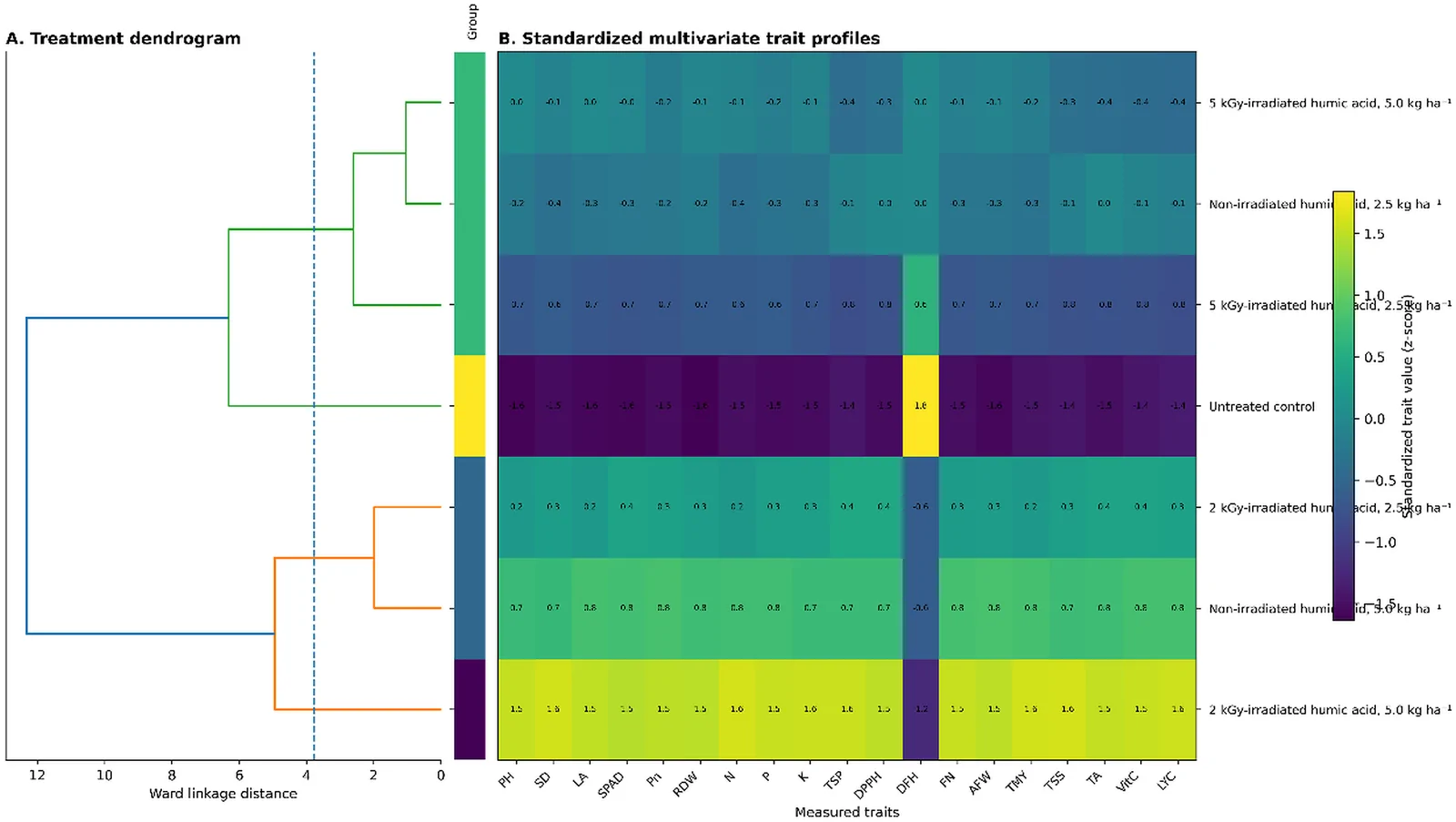
Heatmap showing minimum, mean, and maximum values of individual tomato growth, physiological, nutritional, yield, and fruit quality traits across all treatments. Cell colors represent Z-score–standardized values, while numbers indicate actual trait values.

Hierarchical cluster analysis based on the standardized means of 19 directly measured growth, physiological, nutritional, biochemical, phenological, yield, and fruit-quality traits separated the seven treatments into four biologically interpretable groups (Fig 7). The four-cluster solution provided a parsimonious representation of treatment differentiation, with a mean silhouette coefficient of 0.587 and a cophenetic correlation coefficient of 0.581. Cluster I contained only humic acid irradiated at 2 kGy and applied at 5.0 kg ha^−1^, which was clearly distinguished from the other treatments by consistently high standardized values for vegetative growth, chlorophyll index, net photosynthetic rate, leaf nutrient accumulation, antioxidant-related traits, fruit number, average fruit weight, marketable yield, and fruit-quality attributes, together with fewer days to first harvest. Cluster II comprised non-irradiated humic acid applied at 5.0 kg ha^−1^ and humic acid irradiated at 2 kGy and applied at 2.5 kg ha^−1^; these treatments exhibited broadly similar high-performing multivariate profiles but remained below the superior response observed for humic acid irradiated at 2 kGy and applied at 5.0 kg ha^−1^. Cluster III contained non-irradiated humic acid applied at 2.5 kg ha^−1^ and humic acid irradiated at 5 kGy and applied at either 2.5 or 5.0 kg ha^−1^. These treatments were characterized by intermediate standardized values for most measured traits, indicating that irradiation at 5 kGy did not generate the broad multivariate improvement associated with the 2 kGy dose. The untreated control formed Cluster IV as an independent low-performing group and was separated from all humic-acid-containing treatments by its lower growth, photosynthetic performance, nutrient concentrations, antioxidant capacity, yield components, marketable yield, and fruit-quality values, together with its later first harvest.

### Principal component analysis (PCA)

**Fig 8:**
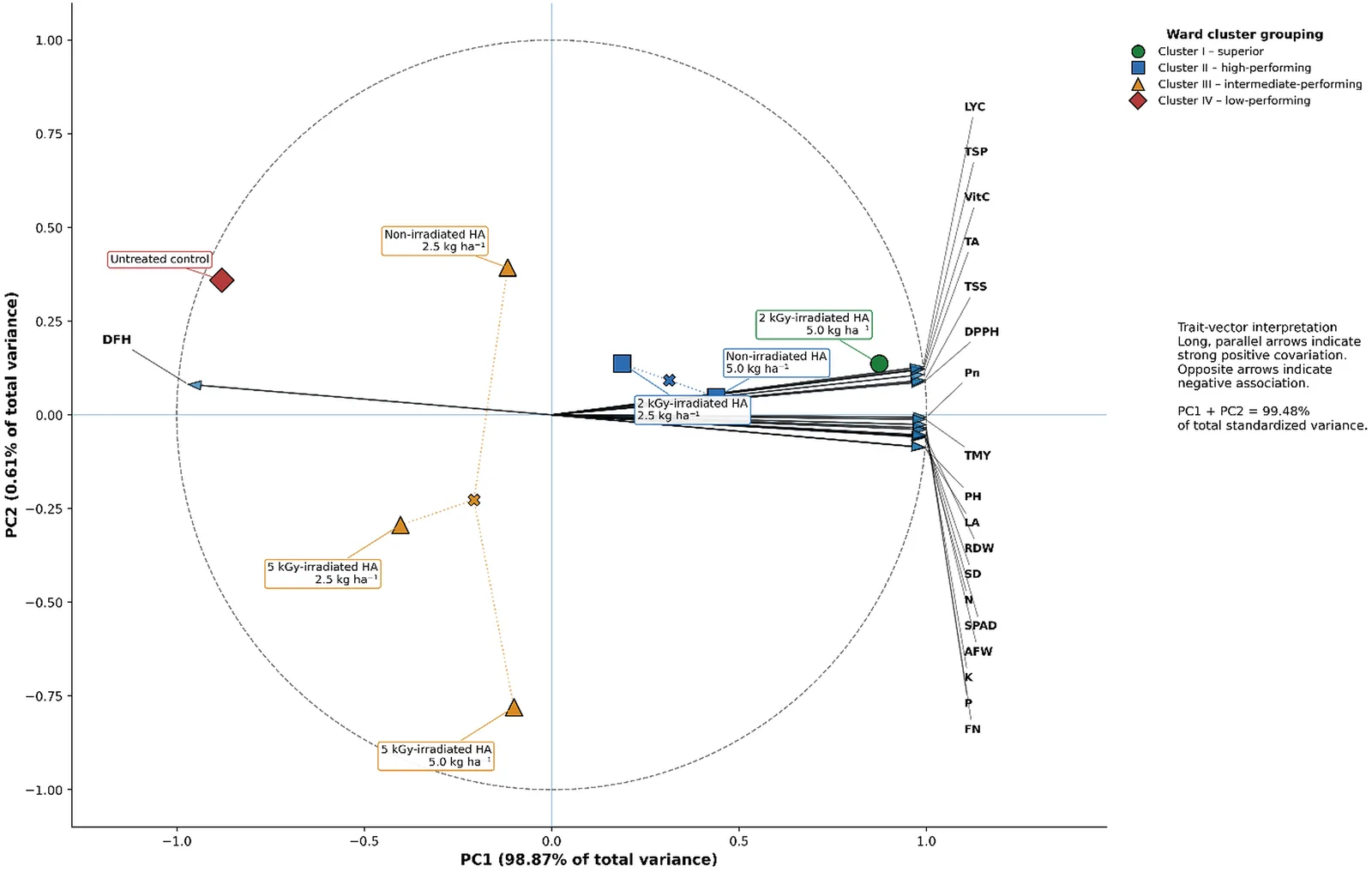
Principal component analysis (PCA) biplot showing the relationships between tomato growth, physiological, nutritional, yield, and fruit quality traits and treatment grouping. Points represent treatments colored according to cluster membership, while vectors indicate trait loadings. The first two principal components explain the majority of the total variance. Principal component analysis was performed on standardized treatment means for 19 directly measured traits. Treatment points are colored and shaped according to the four groups identified by Ward hierarchical cluster analysis: Cluster I, superior; Cluster II, high-performing; Cluster III, intermediate-performing; and Cluster IV, low-performing. Dotted lines connect treatments to their corresponding cluster centroids. Arrows represent correlations between the measured traits and the first two principal components; longer vectors indicate stronger representation in the PC1–PC2 plane, similar vector directions indicate positive associations, and opposing vectors indicate negative associations. PC1 and PC2 explained 98.87% and 0.61% of the total standardized variance, respectively, accounting jointly for 99.48%. Treatment scores were linearly rescaled to the loading space for joint graphical presentation, without altering their relative positions or cluster membership. PH, plant height; SD, stem diameter; LA, leaf area; SPAD, chlorophyll index; Pn, net photosynthetic rate; RDW, root dry weight; N, leaf nitrogen; P, leaf phosphorus; K, leaf potassium; TSP, total soluble phenolics; DPPH, DPPH radical-scavenging activity; DFH, days to first harvest; FN, fruit number per plant; AFW, average fruit weight; TMY, total marketable yield; TSS, total soluble solids; TA, titratable acidity; VitC, vitamin C; and LYC, lycopene.

Principal component analysis of the standardized means of 19 directly measured traits revealed an exceptionally strong common multivariate response among the seven treatments (Fig 8). The first principal component explained 98.87% of the total standardized variance, whereas the second principal component explained 0.61%; together, PC1 and PC2 accounted for 99.48% of the total variation. PC3 explained a further 0.43%, and each remaining component contributed less than 0.04%. The steep decline in explained variance after PC1 indicated that treatment differentiation was governed predominantly by a single integrated performance gradient rather than several independent dimensions. PC1 had strong positive loadings for almost all growth, physiological, nutritional, biochemical, yield, and fruit-quality variables. The largest positive correlations with PC1 were observed for net photosynthetic rate (loading = 0.999), fruit number per plant (0.999), leaf potassium concentration (0.999), leaf phosphorus concentration (0.998), average fruit weight (0.998), root dry weight (0.997), chlorophyll index (0.997), and total marketable yield (0.997). Plant height, stem diameter, leaf area, leaf nitrogen concentration, total soluble phenolics, DPPH radical-scavenging activity, total soluble solids, titratable acidity, vitamin C, and lycopene also had loadings exceeding 0.99 on PC1. In contrast, days to first harvest had a strong negative loading of −0.969. The near-parallel orientation of the positive trait vectors indicated strong coordinated variation among vegetative growth, nutrient status, photosynthetic performance, antioxidant responses, fruit production, marketable yield, and fruit quality. The opposite orientation of days to first harvest demonstrated that treatments associated with stronger growth and productivity also tended to reach marketable harvest earlier.

The distribution of treatment scores along PC1 closely reproduced the four groups identified by Ward hierarchical cluster analysis. Humic acid irradiated at 2 kGy and applied at 5.0 kg ha^−1^, representing the superior Cluster I, had the highest positive PC1 score of 6.639 and was positioned at the positive extreme of the biplot. Its proximity to the vectors for net photosynthetic rate, chlorophyll index, leaf nutrient concentrations, fruit number, average fruit weight, marketable yield, antioxidant activity, and fruit-quality attributes confirmed its consistently favorable performance across the measured trait system. The two treatments assigned to high-performing Cluster II also occupied the positive side of PC1. Non-irradiated humic acid applied at 5.0 kg ha^−1^ had a PC1 score of 3.335, while humic acid irradiated at 2 kGy and applied at 2.5 kg ha^−1^ had a score of 1.431. Their positions between the superior treatment and the intermediate group indicated broadly favorable multivariate responses, although their combined trait performance remained below that of humic acid irradiated at 2 kGy and applied at 5.0 kg ha^−1^. The treatments assigned to intermediate-performing Cluster III occupied negative or near-central positions on PC1. Non-irradiated humic acid applied at 2.5 kg ha^−1^ had a PC1 score of −0.893, humic acid irradiated at 5 kGy and applied at 5.0 kg ha^−1^ had a score of −0.764, and humic acid irradiated at 5 kGy and applied at 2.5 kg ha^−1^ had a score of −3.064. This distribution confirmed that the 5 kGy irradiation dose did not generate the coordinated improvement in growth, photosynthesis, yield, and fruit quality associated with the 2 kGy treatment. Increasing the application rate from 2.5 to 5.0 kg ha^−1^ partly improved the multivariate position of humic acid irradiated at 5 kGy, but it remained within the intermediate cluster.

The untreated control, corresponding to low-performing Cluster IV, had the most negative PC1 score of −6.685 and was clearly separated from all humic-acid-containing treatments. Its position was aligned with the days-to-first-harvest vector and opposite the vectors representing growth, nutrient accumulation, photosynthetic activity, yield components, marketable yield, and fruit quality. This placement reflected its comparatively weak overall performance and later first harvest. Although PC2 contributed only 0.61% of the total variance, it described minor differences in the balance between fruit-quality and vegetative traits. Lycopene, total soluble phenolics, vitamin C, titratable acidity, total soluble solids, and DPPH activity had the largest positive PC2 loadings, ranging from 0.088 to 0.128. Plant height, leaf area, root dry weight, chlorophyll index, stem diameter, and average fruit weight had modest negative PC2 loadings. Accordingly, positive PC2 positions represented slightly greater fruit-quality and antioxidant expression relative to vegetative growth, whereas negative PC2 positions indicated a comparatively stronger vegetative component. However, because PC2 explained less than 1% of the total variance, this secondary pattern should not be given substantial biological weight. All traits were well represented in the PC1–PC2 plane, with combined squared cosine values ranging from 0.946 for days to first harvest to more than 0.999 for several biochemical and fruit-quality traits.

### Multi-criteria decision-making (MCDM) analysis for treatment selection

A comprehensive multi-criteria decision-making analysis was conducted to integrate the simultaneous effects of the treatments on 19 directly measured vegetative, photosynthetic, nutritional, biochemical, phenological, yield, and fruit-quality criteria. Yield increase relative to the control was excluded because it was mathematically derived from total marketable yield and would therefore duplicate information already represented in the decision matrix. Eighteen traits were treated as benefit criteria, for which larger values were considered favorable, whereas days to first harvest was treated as a cost criterion because a lower value indicated earlier production. To avoid disproportionate influence from trait categories containing more variables, the primary weighting system combined balanced functional-category weights with objective CRITIC weights in equal proportions. The resulting hybrid weights are presented in Table 2.

**Table 2:** Decision criteria, optimization directions, and weighting coefficients used in the multi-criteria decision-making analysis. Include the criterion code, complete trait name, functional category, benefit or cost direction, balanced-category weight, CRITIC weight, and final hybrid weight.

Code	Criterion	Category	Direction	Balanced-category weight	CRITIC weight	Final hybrid weight
PH	Plant Height (cm)	Vegetative growth	Benefit	0.0417	0.0470	0.0443
SD	Stem Diameter (mm)	Vegetative growth	Benefit	0.0417	0.0444	0.0430
LA	Leaf Area (cm²/plant)	Vegetative growth	Benefit	0.0417	0.0486	0.0451
SPAD	Chlorophyll Index (SPAD)	Photosynthetic performance	Benefit	0.0833	0.0385	0.0609
Pn	Net Photosynthetic Rate (µmol CO_2_ m^−2^ s^−1^)	Photosynthetic performance	Benefit	0.0833	0.0294	0.0564
RDW	Root Dry Weight (g/plant)	Vegetative growth	Benefit	0.0417	0.0375	0.0396
N	Leaf N (%)	Leaf nutrition	Benefit	0.0556	0.0499	0.0527
P	Leaf P (%)	Leaf nutrition	Benefit	0.0556	0.0334	0.0445
K	Leaf K (%)	Leaf nutrition	Benefit	0.0556	0.0328	0.0442
TSP	Total Soluble Phenolics (mg GAE/g DW)	Antioxidant and biochemical status	Benefit	0.0833	0.0652	0.0743
DPPH	Antioxidant Activity (DPPH, % inhibition)	Antioxidant and biochemical status	Benefit	0.0833	0.0552	0.0693
DFH	Days to First Harvest	Phenology and yield	Cost	0.0417	0.1665	0.1041
FN	Number of Fruits per Plant	Phenology and yield	Benefit	0.0417	0.0327	0.0372
AFW	Average Fruit Weight (g)	Phenology and yield	Benefit	0.0417	0.0362	0.0389
TMY	Total Marketable Yield (kg/plant)	Phenology and yield	Benefit	0.0417	0.0396	0.0407
TSS	Fruit Total Soluble Solids (°Brix)	Fruit quality	Benefit	0.0417	0.0495	0.0456
TA	Fruit Titratable Acidity (% citric acid)	Fruit quality	Benefit	0.0417	0.0611	0.0514
VitC	Vitamin C (mg/100g FW)	Fruit quality	Benefit	0.0417	0.0626	0.0521
LYC	Lycopene (mg/100g FW)	Fruit quality	Benefit	0.0417	0.0698	0.0557

Under the hybrid weighting scheme, phenology and yield accounted for the largest cumulative category weight, representing 22.09% of the total decision importance, followed by fruit quality at 20.48%, vegetative growth at 17.21%, antioxidant and biochemical status at 14.36%, leaf nutrition at 14.14%, and photosynthetic performance at 11.73%. At the individual-criterion level, days to first harvest received the greatest hybrid weight, at 10.41%, reflecting its relatively high dispersion and distinct information content after the cost transformation. Total soluble phenolics and DPPH radical-scavenging activity received weights of 7.43% and 6.93%, respectively, followed by chlorophyll index at 6.09%, net photosynthetic rate at 5.64%, lycopene at 5.57%, leaf nitrogen at 5.27%, and vitamin C at 5.21%. The remaining criteria each contributed between 3.72% and 5.14% of the final decision weight, ensuring that no single growth, yield, or fruit-quality variable exclusively controlled treatment selection (Table 2; S1 Fig).

**Fig 9:**
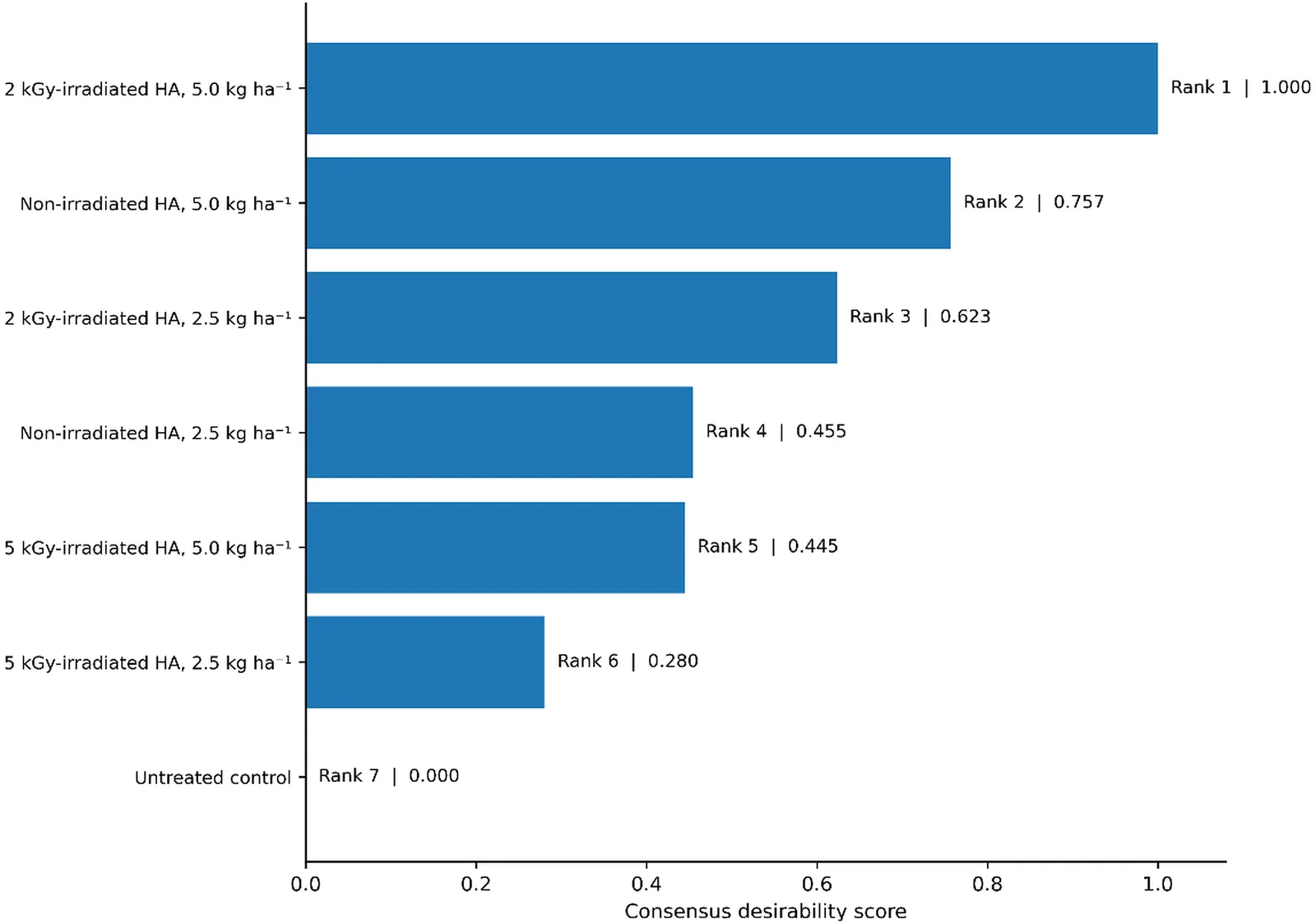
Ensemble multi-criteria decision-making ranking of tomato treatments based on growth, physiological, yield, and fruit-quality criteria. Bars represent the consensus desirability score obtained by averaging normalized TOPSIS, VIKOR, WASPAS, and EDAS scores under the hybrid balanced-category–CRITIC weighting scheme. Higher scores indicate more favorable integrated treatment performance. Numbers beside the bars indicate the final consensus rank.

**Fig 10:**
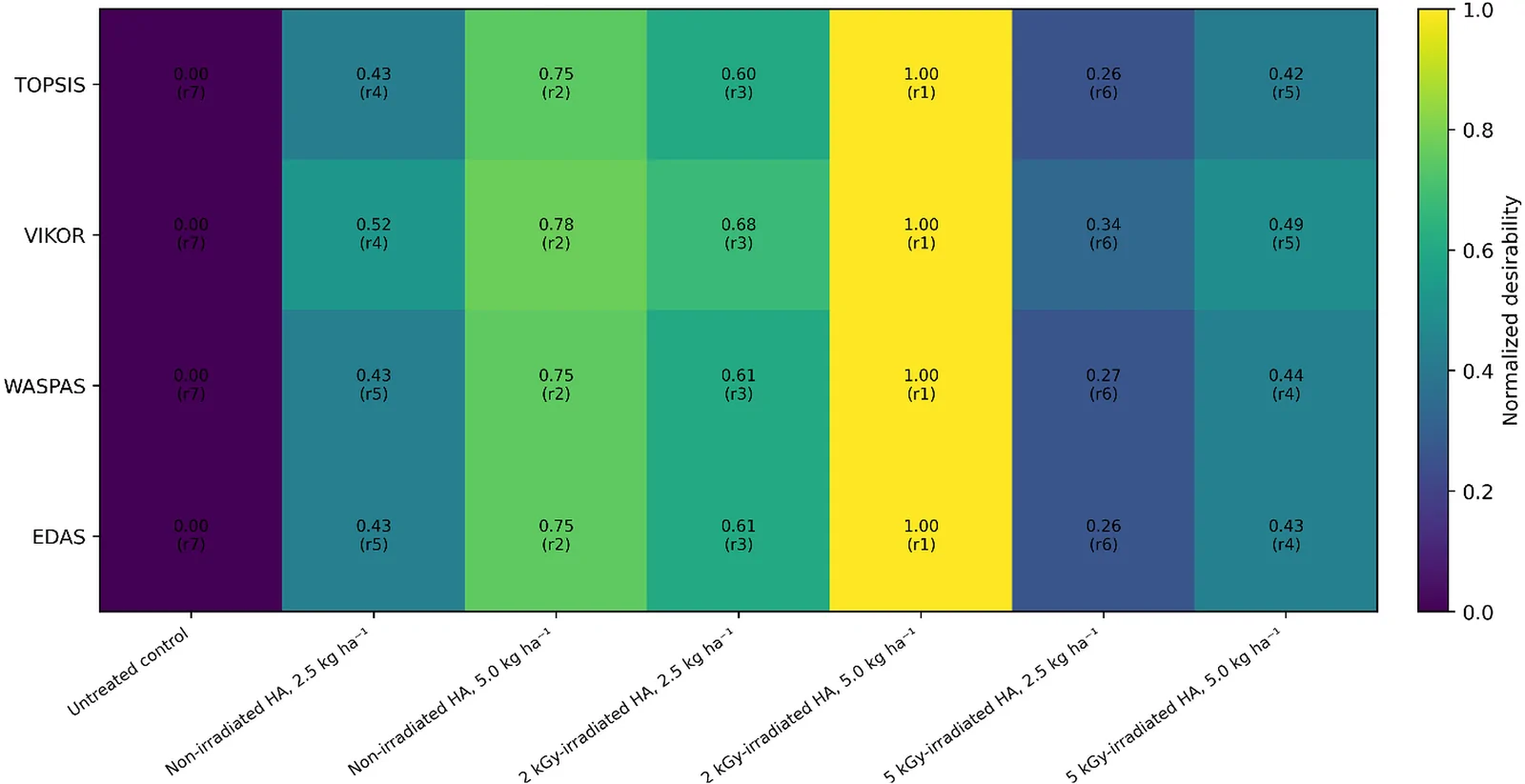
Concordance of TOPSIS, VIKOR, WASPAS, and EDAS evaluations of tomato treatments. The heatmap presents method-specific normalized desirability scores. Values in parentheses indicate the rank assigned by each method. Higher normalized values and lower rank numbers indicate more favorable treatments.

The TOPSIS, VIKOR, WASPAS, and EDAS procedures produced highly concordant treatment rankings (Table 3; Figs 9 and 10). Humic acid irradiated at 2 kGy and applied at 5.0 kg ha^−1^ was ranked first by all four methods. This treatment obtained the maximum TOPSIS relative-closeness coefficient of 1.000, the maximum VIKOR desirability of 1.000, corresponding to Q = 0.000, the maximum WASPAS score of 1.000, and the maximum EDAS appraisal score of 1.000. Its normalized ensemble desirability was consequently 1.000, with an average method rank of 1.0 and the maximum Borda total of 28 points. The treatment therefore represented the positive ideal solution across the combined growth, physiological, nutritional, yield, earliness, and fruit-quality criteria.

**Table 3:** Method-specific and consensus ranking of tomato treatments using TOPSIS, VIKOR, WASPAS, and EDAS. Report each method’s score and rank, average method rank, consensus desirability score, and final consensus rank.

Treatment	TOPSIS	VIKOR	WASPAS	EDAS	Average rank	Consensus score	Consensus rank
2 kGy-irradiated humic acid, 5.0 kg ha^−1^	1.000 (1)	1.000 (1)	1.000 (1)	1.000 (1)	1.000	1.000	1
Non-irradiated humic acid, 5.0 kg ha^−1^	0.746 (2)	0.777 (2)	0.929 (2)	0.751 (2)	2.000	0.757	2
2 kGy-irradiated humic acid, 2.5 kg ha^−1^	0.602 (3)	0.676 (3)	0.887 (3)	0.607 (3)	3.000	0.623	3
Non-irradiated humic acid, 2.5 kg ha^−1^	0.429 (4)	0.524 (4)	0.837 (5)	0.432 (5)	4.500	0.455	4
5 kGy-irradiated humic acid, 5.0 kg ha^−1^	0.420 (5)	0.490 (5)	0.838 (4)	0.434 (4)	4.500	0.445	5
5 kGy-irradiated humic acid, 2.5 kg ha^−1^	0.255 (6)	0.338 (6)	0.789 (6)	0.264 (6)	6.000	0.280	6
Untreated control	0.000 (7)	0.000 (7)	0.713 (7)	0.000 (7)	7.000	0.000	7

The superiority of humic acid irradiated at 2 kGy and applied at 5.0 kg ha^−1^ was also supported by the VIKOR compromise conditions. The difference between the Q values of the first- and second-ranked treatments was 0.223, exceeding the acceptable-advantage threshold of 0.167 calculated as 1/(7 − 1). In addition, this treatment had the lowest group-utility measure and the lowest maximum individual regret. Thus, its selection was supported by both overall criterion performance and the absence of a major weakness in any individual criterion.

Pareto-dominance analysis provided additional support for the first-ranked treatment. Humic acid irradiated at 2 kGy and applied at 5.0 kg ha^−1^ dominated all six remaining alternatives after conversion of the decision criteria to a common benefit orientation. It was not dominated by any other treatment. This result demonstrated that no competing treatment improved one or more criteria without simultaneously declining in at least one other criterion relative to the first-ranked alternative.

Non-irradiated humic acid applied at 5.0 kg ha^−1^ was ranked second by every MCDM method. It obtained a TOPSIS score of 0.746, VIKOR desirability of 0.777, WASPAS score of 0.929, and EDAS score of 0.751. Its consensus desirability was 0.757, with an average method rank of 2.0. This treatment dominated five of the six other alternatives but was itself dominated by humic acid irradiated at 2 kGy and applied at 5.0 kg ha^−1^. Its consistently high position indicated that increasing the application rate of non-irradiated humic acid to 5.0 kg ha^−1^ generated a broad improvement across the decision criteria, although it remained inferior to the corresponding 2 kGy-irradiated treatment. Humic acid irradiated at 2 kGy and applied at 2.5 kg ha^−1^ was ranked third by all four methods. Its TOPSIS, VIKOR, WASPAS, and EDAS scores were 0.602, 0.676, 0.887, and 0.607, respectively, resulting in a consensus desirability of 0.623. This treatment dominated the untreated control, non-irradiated humic acid applied at 2.5 kg ha^−1^, and both treatments containing humic acid irradiated at 5 kGy. Its stable third-place ranking showed that moderate irradiation remained favorable even at the lower application rate, but that increasing the application rate to 5.0 kg ha^−1^ was necessary to obtain the maximum integrated response.

The fourth and fifth positions showed the only meaningful disagreement among the four ranking methods. Non-irradiated humic acid applied at 2.5 kg ha^−1^ was ranked fourth by TOPSIS and VIKOR but fifth by WASPAS and EDAS. Conversely, humic acid irradiated at 5 kGy and applied at 5.0 kg ha^−1^ was ranked fifth by TOPSIS and VIKOR but fourth by WASPAS and EDAS. Both treatments therefore had an average method rank of 4.5 and an equal Borda total of 14 points. The final consensus ranking placed non-irradiated humic acid applied at 2.5 kg ha^−1^ in fourth position because its ensemble desirability of 0.455 was slightly higher than the value of 0.445 obtained for humic acid irradiated at 5 kGy and applied at 5.0 kg ha^−1^. The small difference between these alternatives indicates that they should be interpreted as a closely matched intermediate-priority pair rather than as treatments with strongly distinct overall performance.

Humic acid irradiated at 5 kGy and applied at 2.5 kg ha^−1^ ranked sixth under all four MCDM procedures. Its TOPSIS score was 0.255, VIKOR desirability was 0.338, WASPAS score was 0.789, and EDAS score was 0.264, producing a consensus desirability of 0.280. Although this treatment remained superior to the untreated control, it was dominated by every other humic-acid-containing treatment. The result confirms that the combination of the higher irradiation dose and lower application rate was the least favorable humic acid treatment when the complete trait system was considered. The untreated control occupied the seventh and final position under every ranking method. It had TOPSIS, VIKOR-desirability, and EDAS scores of 0.000 and the lowest WASPAS score of 0.713. After method-specific normalization, its consensus desirability was 0.000. The control was dominated by all six humic-acid-containing treatments, reflecting its lower growth, photosynthetic activity, nutrient accumulation, antioxidant response, yield, and fruit-quality performance, together with its later first harvest. Agreement among the four MCDM algorithms was exceptionally high. Spearman rank correlations ranged from 0.964 to 1.000. TOPSIS and VIKOR generated identical treatment orders, while WASPAS and EDAS also produced identical orders. The only difference between these two pairs of methods concerned the closely matched fourth- and fifth-ranked treatments. The leading three positions and the two lowest positions were invariant across all methods, demonstrating that the principal treatment-selection conclusion was not dependent on the mathematical structure of a single MCDM algorithm (Fig 10).

**Fig 11:**
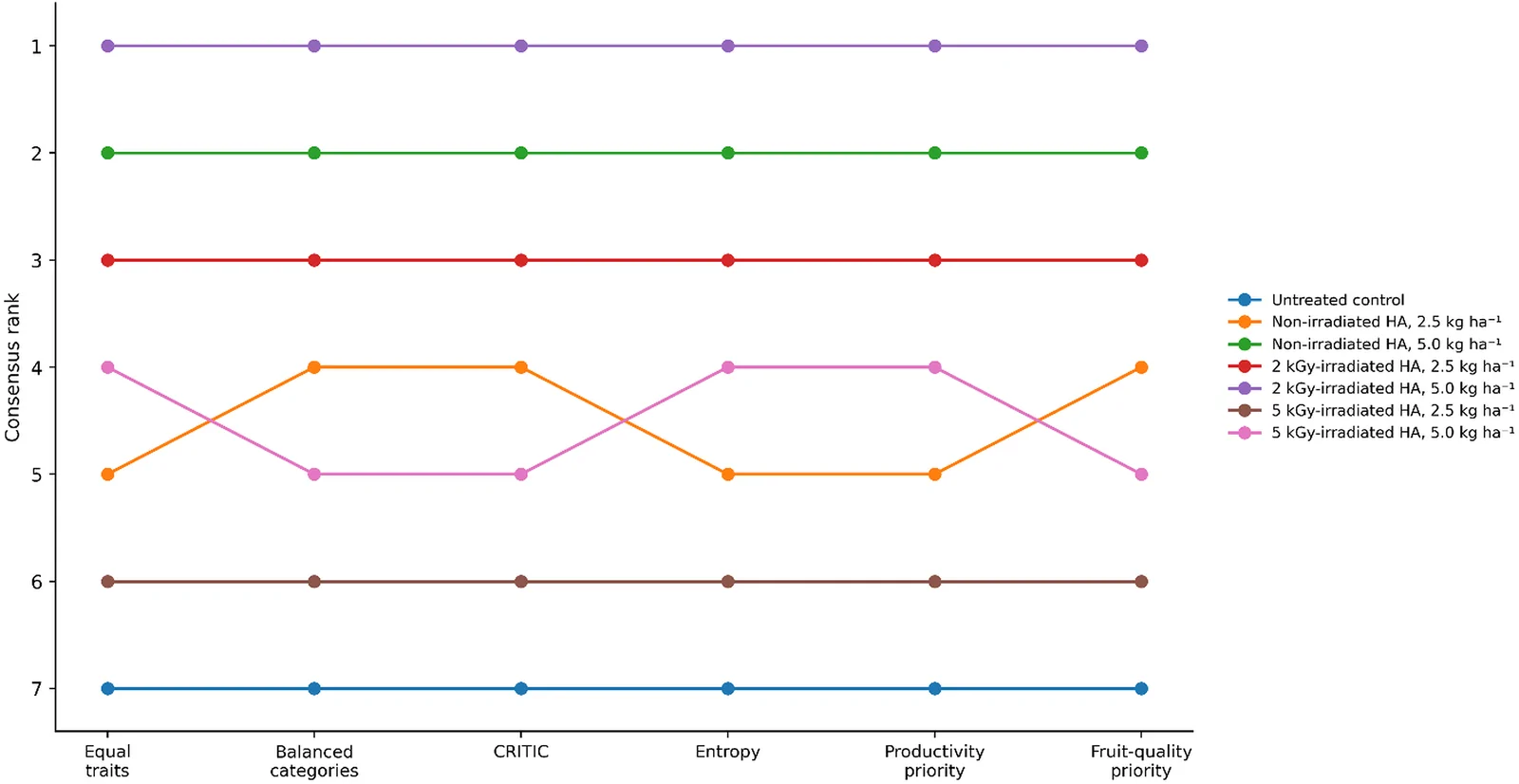
Sensitivity of consensus treatment rankings to alternative criterion-weighting scenarios. Treatment ranks were recalculated using equal-trait, balanced-category, CRITIC, entropy, productivity-priority, and fruit-quality-priority weights. Rank 1 indicates the preferred treatment. Each scenario represents the consensus of TOPSIS, VIKOR, WASPAS, and EDAS.

Sensitivity analysis further demonstrated the robustness of the treatment ranking to alternative assumptions regarding criterion importance (Table 4; Fig 11). Six weighting scenarios were evaluated: equal weights for all traits, equal weights for the six functional categories, CRITIC objective weights, entropy objective weights, productivity-priority weights, and fruit-quality-priority weights. Humic acid irradiated at 2 kGy and applied at 5.0 kg ha^−1^ ranked first under all six scenarios. Non-irradiated humic acid applied at 5.0 kg ha^−1^ remained second, and humic acid irradiated at 2 kGy and applied at 2.5 kg ha^−1^ remained third in every scenario. Humic acid irradiated at 5 kGy and applied at 2.5 kg ha^−1^ consistently ranked sixth, while the untreated control consistently ranked seventh.

**Table 4:** Sensitivity of consensus treatment rankings to alternative criterion-weighting scenarios. Include ranks under equal-trait, balanced-category, CRITIC, entropy, productivity-priority, and fruit-quality-priority weighting, together with mean and range of ranks.

Treatment	Equal traits	Balanced categories	CRITIC	Entropy	Productivity priority	Fruit-quality priority	Mean rank	Rank range
Untreated control	7	7	7	7	7	7	7.00	7–7
Non-irradiated humic acid, 2.5 kg ha^−1^	5	4	4	5	5	4	4.50	4–5
Non-irradiated humic acid, 5.0 kg ha^−1^	2	2	2	2	2	2	2.00	2–2
2 kGy-irradiated humic acid, 2.5 kg ha^−1^	3	3	3	3	3	3	3.00	3–3
2 kGy-irradiated humic acid, 5.0 kg ha^−1^	1	1	1	1	1	1	1.00	1–1
5 kGy-irradiated humic acid, 2.5 kg ha^−1^	6	6	6	6	6	6	6.00	6–6
5 kGy-irradiated humic acid, 5.0 kg ha^−1^	4	5	5	4	4	5	4.50	4–5

**Fig 12:**
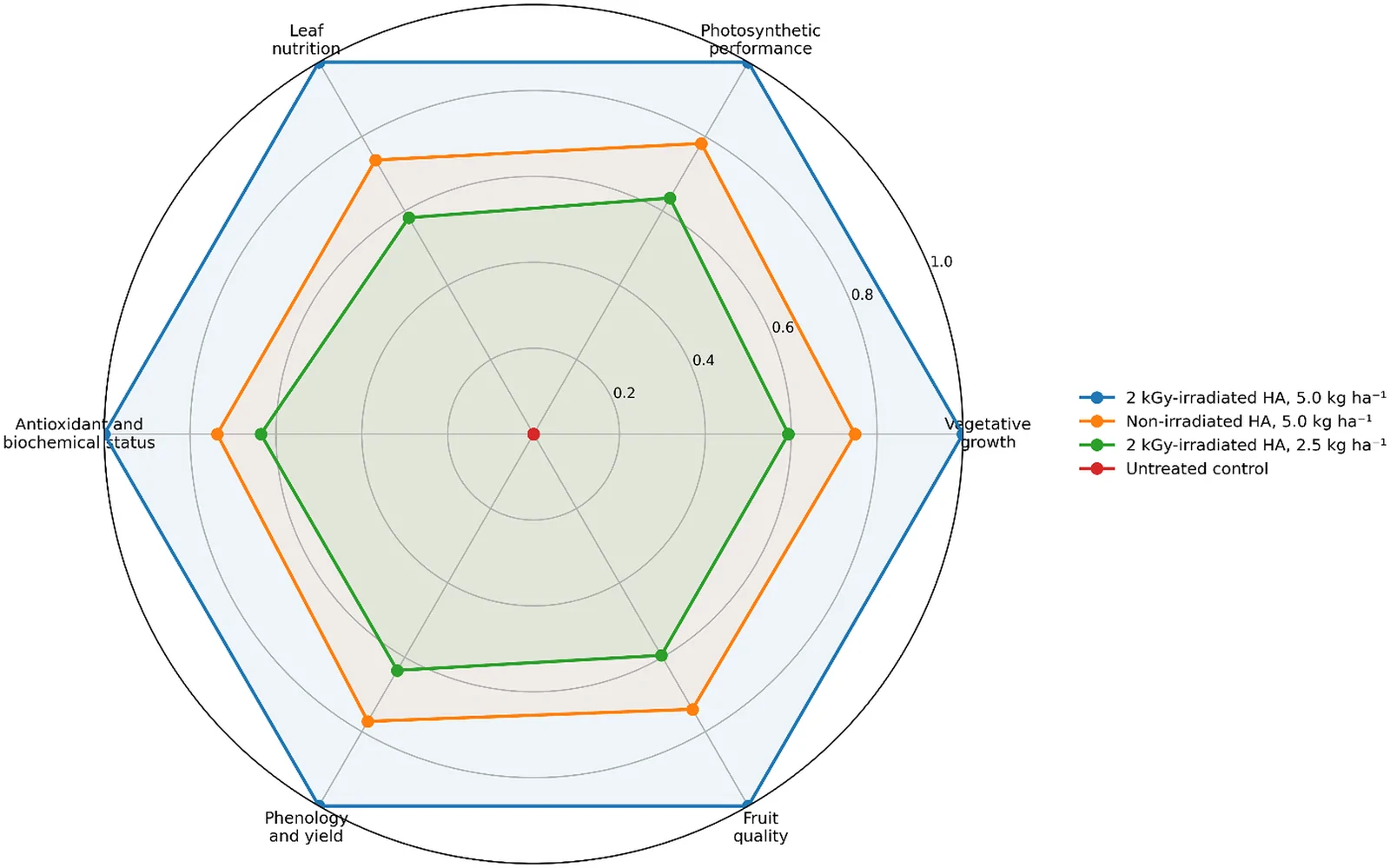
Functional-category performance profiles of the three highest-ranked tomato treatments and the untreated control. Scores represent the mean benefit-oriented min–max-normalized values of the criteria assigned to vegetative growth, photosynthetic performance, leaf nutrition, antioxidant and biochemical status, phenology and yield, and fruit quality.

Only the fourth and fifth positions were sensitive to the weighting strategy. Non-irradiated humic acid applied at 2.5 kg ha^−1^ ranked fourth under balanced-category, CRITIC, and fruit-quality-priority weighting but ranked fifth under equal-trait, entropy, and productivity-priority weighting. Humic acid irradiated at 5 kGy and applied at 5.0 kg ha^−1^ showed the inverse pattern. This limited rank exchange did not affect selection of the preferred treatment or identification of the three highest-performing alternatives. Category-level performance profiles were consistent with the consensus MCDM ranking (Fig 12). Humic acid irradiated at 2 kGy and applied at 5.0 kg ha^−1^ achieved the maximum normalized value of 1.000 in all six functional categories. Non-irradiated humic acid applied at 5.0 kg ha^−1^ produced category scores ranging from 0.737 for leaf nutrition and antioxidant-related performance to 0.781 for photosynthetic performance. Humic acid irradiated at 2 kGy and applied at 2.5 kg ha^−1^ produced category values ranging from 0.582 for leaf nutrition to 0.635 for photosynthetic performance, antioxidant-related traits, and phenology and yield. In contrast, the untreated control represented the minimum reference value in every category.

## Discussion

Fourier-transform infrared spectroscopy indicated that gamma irradiation modified the humic acid without eliminating its principal functional-group regions. The broad absorption band remained within 3432–3440 cm^−1^ in the non-irradiated and irradiated samples. This region is generally attributed to hydrogen-bonded O–H stretching in alcoholic, phenolic, and carboxylic groups, with a possible contribution from N–H stretching. Similarly, the prominent band observed at 1638.48 cm^−1^ in the non-irradiated material shifted slightly to 1636.53 and 1634.69 cm^−1^ after irradiation at 2 and 5 kGy, respectively. In heterogeneous humic materials, absorption within this region may arise from overlapping aromatic C = C stretching, conjugated carbonyl groups, ionized carboxyl groups, and bound water rather than from a single discrete functional group [19–21]. Bands within the C–O-rich fingerprint region, particularly near 1156 and 1110–1113 cm^−1^, also remained detectable after irradiation. Their persistence suggests that alcohol-, phenol-, ether-, or carbohydrate-associated C–O structures remained present at both irradiation doses. These observations collectively indicate retention of the broad functional framework of the commercial humic material, accompanied by dose-dependent changes in hydrogen bonding and in the local chemical environments of aromatic, carbonyl, carboxylate, and C–O-containing structures. The spectrum obtained after irradiation at 2 kGy exhibited resolved bands at approximately 2923.79 and 2858.10 cm^−1^, which are commonly assigned to asymmetric and symmetric C–H stretching of aliphatic CH_2_ and CH_3_ groups, respectively. The 5 kGy spectrum, in contrast, showed an additional resolved band at 1387.69 cm^−1^ and a modified low-wavenumber fingerprint profile. Absorption near 1380–1400 cm^−1^ in humic materials may include contributions from symmetric carboxylate stretching, phenolic O–H deformation, and aliphatic C–H deformation. Because humic acids are chemically heterogeneous and their infrared bands are broad and strongly overlapping, these assignments should be considered tentative rather than diagnostic of individual molecular structures [19-21]. The different spectral profiles obtained at 2 and 5 kGy demonstrate that the two irradiation treatments did not produce identical physicochemical effects. Previous studies have similarly shown that the influence of gamma irradiation on humic substances is dose dependent and may involve changes in functional-group abundance, molecular-size distribution, carbon allocation among molecular fractions, and metal-complexation behavior [22,23]. Zhao et al. [23], for example, demonstrated through sequential ultrafiltration, total organic carbon analysis, and potentiometric titration that gamma irradiation redistributed humic material toward smaller molecular-weight fractions and altered the relative abundance of carboxylic and phenolic groups. However, that investigation used humic acid in aqueous solution and included irradiation doses extending to 100 kGy; its results therefore provide mechanistic context but cannot be transferred directly to the dry commercial powder and low doses used in the present experiment. The greater plant response obtained with the 2 kGy material may consequently have been associated with a physicochemical modification that preserved biologically relevant functional domains while altering their accessibility or chemical environment. Conversely, the weaker response at 5 kGy may reflect a qualitatively different or more extensive modification of the humic material. This interpretation is consistent with the observed dose-dependent FTIR differences and with previous evidence that irradiation dose influences humic-substance composition and complexation behavior. Nevertheless, it remains a hypothesis because FTIR band shifts alone cannot establish which chemical changes were responsible for the biological response. Direct relationships among FTIR features, root activity, nutrient behavior, and crop performance were not tested in the present study. The FTIR findings must also be interpreted cautiously because relative band intensities can be affected by sample preparation and measurement conditions. Depending on the analytical mode, variation may arise from sample concentration, baseline treatment, potassium-bromide dilution and pellet thickness, or differences in contact pressure between the sample and the attenuated-total-reflectance crystal. Experimental comparisons have shown that both sample-preparation procedures and measurement methodology can materially affect ATR–FTIR spectral profiles [24]. Therefore, unless independently prepared analytical replicates and standardized acquisition conditions were used, emphasis should be placed primarily on reproducible band positions and broad spectral patterns rather than on small differences in relative intensity. Moreover, FTIR does not directly quantify molecular-weight distribution and cannot, on its own, demonstrate specific bond cleavage, molecular fragmentation, or a reduction in average molecular size. Such conclusions require complementary separation and characterization methods. Sequential ultrafiltration has been used to quantify the distribution of irradiated humic acid among defined molecular-size fractions, whereas size-exclusion or gel-permeation chromatography can provide continuous apparent molecular-weight profiles. Potentiometric titration, elemental analysis, nuclear magnetic resonance spectroscopy, X-ray photoelectron spectroscopy, viscosity measurements, and direct functional-group quantification can provide additional evidence regarding changes in acidity, elemental composition, structural domains, and aggregation behavior [21,23]. Accordingly, the present FTIR results demonstrate dose-dependent spectroscopic modification but do not establish molecular fragmentation or molecular-weight reduction.

The present study demonstrated that gamma irradiation altered the biological effectiveness of humic acid in a dose-dependent manner. Across nearly all measured variables, humic acid irradiated at 2 kGy and applied at 5.0 kg ha^−1^ produced the most favorable response, whereas irradiation at 5 kGy generally reduced the benefit obtained from increasing the application rate. This treatment hierarchy was evident in vegetative growth, chlorophyll status, net photosynthetic rate, leaf nutrient concentrations, phenolic accumulation, antioxidant capacity, earliness, yield components, marketable yield, and fruit quality, indicating that the 2 kGy treatment improved coordinated whole-plant performance rather than a single isolated trait. However, 2 kGy should not be considered universally optimal because humic substances differ substantially in origin, molecular composition, molecular-size distribution, functional groups, water content, pH, and physical form, all of which can influence their physicochemical and radiolytic responses [19]. The present results identify 2 kGy only as the most effective of the two irradiation doses evaluated under these experimental conditions. A broader dose–response study incorporating additional irradiation levels and direct physicochemical characterization would be required to define the true optimum. The significant irradiation dose × humic acid rate interaction for total marketable yield provided the clearest evidence that the response to humic acid rate depended on the preceding irradiation treatment. Increasing the rate from 2.5 to 5.0 kg ha^−1^ significantly increased yield when the humic acid was non-irradiated or irradiated at 2 kGy, but not when irradiated at 5 kGy. This pattern is consistent with a hormetic-like response in which moderate physicochemical modification enhances biological activity, whereas stronger modification becomes less effective; previous studies have similarly demonstrated dose-dependent alterations in humic-acid properties following gamma irradiation [17].

Earlier irradiation studies have shown that gamma exposure can alter the molecular-weight distribution, carbon content, functional-group composition, and complexation behavior of humic substances. Zhao et al. reported gamma-induced degradation of humic acid and redistribution of functional groups among molecular-size fractions, while Sasaki et al. demonstrated that irradiation effects depend on dose, dose rate, and the nature of the humic material. These findings provide a plausible physicochemical context for the superior response at 2 kGy and the attenuation observed at 5 kGy. However, because the irradiated products used in the present experiment were not characterized directly, molecular fragmentation, oxidation, or functional-group exposure remain mechanistic hypotheses rather than demonstrated explanations [22,23,25]. A nonlinear biological response to humic-derived materials has also been reported in tomato. Suh et al. [26] found that moderate fulvic acid concentrations increased tomato biomass and marketable yield, whereas the highest tested concentration reduced growth and produced smaller fruits. Although that study used foliar fulvic acid rather than gamma-irradiated soil-applied humic acid, the response supports the broader principle that humic-substance effectiveness depends on dose and that increasing the amount or intensity of treatment does not necessarily produce progressively greater benefits [26].

Humic acid irradiated at 2 kGy and applied at 5.0 kg ha^−1^ increased plant height by approximately 39%, leaf area by 56%, and root dry weight by 77% relative to the untreated control. Stem diameter also increased, indicating that the treatment promoted both longitudinal and radial shoot development. These traits are not interchangeable. Plant height reflects axial growth, stem diameter indicates structural support and vascular development, leaf area defines the size of the photosynthetically active canopy, and root dry weight represents belowground biomass investment. Their simultaneous improvement therefore indicates a balanced stimulation of both shoot and root systems rather than excessive elongation alone. The increase in root dry weight is particularly relevant because the humic acid was applied as a soil drench. Root development determines the volume of substrate explored and affects the plant’s capacity to access water and nutrients. Experimental studies with humic acids have demonstrated stimulation of root elongation, lateral-root emergence, and plasma-membrane H ⁺ -ATPase activity, while other work has linked humic-substance-induced lateral-root formation to auxin-related signaling. These mechanisms could contribute to the greater root biomass observed here, but they were not measured directly and should not be presented as confirmed processes in the present tomato plants [27,28]. The positive growth response agrees with earlier tomato research showing that commercial humic acid can modify plant growth and mineral nutrition. It is also consistent with the study of Suh et al.[26], in which an appropriate fulvic acid concentration increased plant height and dry biomass. The comparatively weak response to humic acid irradiated at 5 kGy and applied at 2.5 kg ha^−1^ further emphasizes that growth promotion depended on both irradiation dose and application rate [26,29].

The increases in chlorophyll index and net photosynthetic rate provide a physiological link between enhanced vegetative growth and reproductive productivity. Relative to the control, the leading treatment increased SPAD by approximately 33% and net photosynthetic rate by 48%. SPAD is a nondestructive indicator of leaf greenness and is commonly used as a proxy for chlorophyll and crop nitrogen status [30]. Net photosynthetic rate more directly represents the instantaneous capacity of leaves to assimilate CO_2_ under the specified measurement conditions. Their concurrent enhancement indicates that the larger canopy was also physiologically more active. The correlation network supported this interpretation: net photosynthetic rate was positively associated with fruit number and marketable yield, while leaf area was associated with chlorophyll index and yield. Marketable yield was also associated with average fruit weight, indicating that increased productivity resulted from improvements in both fruit number and fruit size. The strong association between net photosynthetic rate and fruit number is physiologically relevant because flowering, fruit set, and fruit growth impose substantial carbon demand, and photosynthetic carbon supply contributes to source–sink partitioning and yield formation in tomato [31]. However, these relationships were correlational, and net photosynthesis was measured at only one developmental stage; therefore, they indicate coordinated variation between source activity and reproductive output rather than a direct causal pathway. Previous studies similarly showed that effective humic or fulvic acid treatments increased tomato growth, fruit number, marketable yield, and fruit quality [26,32].

The 2 kGy–5.0 kg ha^−1^ treatment increased leaf nitrogen, phosphorus, and potassium concentrations by approximately 35%, 63%, and 54%, respectively, relative to the control. These macronutrients have distinct physiological roles: nitrogen is a major constituent of chlorophyll, photosynthetic proteins, enzymes, and nucleic acids; phosphorus participates in ATP-dependent metabolism, phosphorylation, membrane structure, and assimilate partitioning; and potassium contributes to osmotic regulation, enzyme activation, stomatal function, charge balance, and phloem transport. Their concurrent enrichment is therefore consistent with the observed increases in chlorophyll index, net photosynthetic rate, canopy development, and yield, although the present data do not establish direct causation. Earlier tomato studies similarly showed that commercial humic acid can modify plant mineral nutrition, while moderate fulvic acid application increased leaf phosphorus and calcium, although responses varied among nutrients [26,29]. These findings indicate that nutrient responses to humic substances depend on product composition, application rate, delivery method, substrate conditions, and the specific element considered.

The treatment-adjusted network provided a more selective interpretation than treatment-mean comparisons alone. Leaf potassium was positively associated with plant height and leaf area, while phosphorus was positively associated with potassium. Leaf nitrogen, however, did not retain a correlation at the selected network threshold after treatment effects were removed. Thus, although the treatments increased the mean concentrations of all three nutrients, only some nutrient–growth relationships remained strong at the replicate level, preventing direct attribution of every growth response to all measured elements. The results should therefore be described as changes in leaf nutrient status rather than total nutrient uptake. Organ nutrient accumulation is generally estimated by multiplying nutrient concentration by organ dry biomass, and whole-plant accumulation requires summation across roots, stems, leaves, and fruits [33,34]. Leaf concentration alone may also be affected by nutrient acquisition, transport, retranslocation, tissue partitioning, and biomass dilution [33]. Consequently, the present data cannot establish whole-plant nutrient accumulation, nutrient-use efficiency, or elemental balance. Humic acid may influence both soil nutrient availability and plant physiological processes, but these pathways cannot be separated without genuine initial and replicated post-harvest substrate analyses [35,36].

The leading treatment increased total soluble phenolics by approximately 69% and DPPH radical-scavenging activity by 31% relative to the control. Total soluble phenolics estimate the pool of methanol-extractable phenolic compounds, whereas the DPPH assay measures the capacity of an extract to neutralize a stable synthetic radical under in vitro conditions. The parallel increases suggest that greater phenolic accumulation contributed, at least partly, to the enhanced radical-scavenging capacity of the leaf extracts. Previous tomato studies have shown that humic- and fulvic-acid-containing preharvest treatments can improve carotenoid, soluble-solids, and ascorbic-acid concentrations, while an organo-mineral fertilizer containing humic acid improved yield and antioxidant-related responses under salinity [37,38]. These findings support the capacity of humic substances to influence antioxidant-associated metabolism, although the treatment formulations and environmental conditions differed from those used here. However, DPPH activity should not be equated with reduced oxidative damage in living tissues because it is an extract-based chemical assay and does not measure intracellular reactive oxygen species, membrane integrity, or antioxidant-enzyme activity [39]. PAL, SOD, CAT, peroxidases, ascorbate peroxidase, glutathione reductase, MDA, hydrogen peroxide, superoxide, and electrolyte leakage were not measured. Therefore, the present study demonstrates increased total soluble phenolics and in vitro radical-scavenging capacity but cannot establish activation of enzymatic antioxidant defenses or reduced lipid peroxidation. Likewise, the increase in phenolics is compatible with altered phenylpropanoid metabolism, but PAL activity and related gene expression were not determined. Future studies should combine targeted phenolic profiling with PAL activity, antioxidant enzymes, redox metabolites, and oxidative-damage indicators.

Humic acid irradiated at 2 kGy and applied at 5.0 kg ha^−1^ advanced the first marketable harvest by approximately five days relative to the control. Days to first harvest was the most highly connected node in the treatment-adjusted network and was negatively associated with leaf area, plant height, stem diameter, root dry weight, net photosynthetic rate, fruit number, and marketable yield. Thus, earlier production was associated with stronger vegetative development, greater physiological activity, and higher reproductive output rather than with a yield penalty. Earlier harvest has practical agronomic value because it can extend the effective marketing period and potentially improve economic return in protected tomato production. This response is biologically plausible because rapid canopy establishment, improved nutrient status, and stronger carbon assimilation may accelerate the accumulation of resources required for flowering, fruit development, and maturation. Previous greenhouse research similarly showed that biostimulant application advanced tomato flowering and fruit set while increasing marketable yield [40]. Nevertheless, flowering date, fruit-set dynamics, thermal time, and fruit-development duration were not measured separately; therefore, the precise developmental basis of the earlier harvest remains unresolved. The highest-yielding treatment produced approximately 54% more fruits, 23% heavier fruits, and 90% greater marketable yield than the untreated control, demonstrating that the yield response resulted from simultaneous improvements in fruit number and fruit size. Humic-acid-containing organo-mineral treatments have likewise increased tomato marketable yield, with yield responses associated with improved leaf greenness and crop performance [32]. The interaction analysis further showed that the yield benefit of increasing the humic acid rate was greatest at 2 kGy, remained substantial without irradiation, and was attenuated at 5 kGy. Thus, irradiation modified the response to humic acid rate rather than exerting a constant additive effect.

The 2 kGy–5.0 kg ha^−1^ treatment also produced the highest total soluble solids, titratable acidity, vitamin C, and lycopene. Compared with the control, total soluble solids increased by approximately 31%, vitamin C by 39%, and lycopene by 60%. These variables represent different aspects of fruit quality. Total soluble solids contribute to sweetness and processing value; titratable acidity influences flavor balance; vitamin C contributes to nutritional and antioxidant value; and lycopene is the principal red carotenoid associated with tomato color and nutritional quality. The concurrent increase in yield and fruit-quality variables indicates that the leading treatment did not produce an evident dilution trade-off. Instead, greater productivity was accompanied by higher concentrations of several quality-related compounds. Net photosynthetic rate was positively associated with lycopene, and fruit number was also positively related to lycopene in the treatment-adjusted network. These relationships suggest that greater reproductive output did not occur at the expense of lycopene concentration within the tested treatment range. Earlier tomato studies support the potential of humic substances to improve fruit composition. Preharvest humic and fulvic acid treatments have increased carotenoids, soluble solids, ascorbic acid, and other quality attributes, and soil or foliar humic-containing fertilization has been reported to influence tomato productivity and fruit characteristics [32,37]. The mechanism underlying these fruit-quality responses remains uncertain. Improved photosynthetic activity could increase carbohydrate availability and metabolic precursor supply, while improved potassium status could support assimilate transport and fruit metabolism. However, fruit sugar composition, organic-acid metabolism, phloem transport, carotenoid-biosynthesis enzymes, and relevant gene-expression responses were not determined. The observed relationships should therefore be interpreted as coordinated physiological outcomes rather than as evidence for a defined biochemical pathway.

The hierarchical cluster analysis separated the treatments into four biologically interpretable groups. Humic acid irradiated at 2 kGy and applied at 5.0 kg ha^−1^ formed a distinct superior cluster, while non-irradiated humic acid at 5.0 kg ha^−1^ and 2 kGy-irradiated humic acid at 2.5 kg ha^−1^ formed the next performance tier. The untreated control formed an independent low-performing group. This structure indicates that treatment differentiation was consistent across the 19 measured traits rather than being driven exclusively by yield. PCA produced the same treatment continuum, with PC1 explaining 98.87% of the standardized variance and showing positive associations with nearly all favorable traits but a negative association with days to first harvest. The unusually high variance explained by PC1 should be interpreted cautiously because the analysis was based on only seven treatment means and most traits followed a similar treatment-performance gradient. Under strong collinearity, PCA can concentrate most variation in the first component; however, with few observations relative to the number of variables, the resulting structure should be considered exploratory rather than independent evidence of a physiological mechanism [41]. The ensemble MCDM analysis reinforced the same ranking: TOPSIS, VIKOR, WASPAS, and EDAS consistently selected the 2 kGy–5.0 kg ha^−1^ treatment as the best alternative, followed by non-irradiated humic acid at 5.0 kg ha^−1^ and 2 kGy-irradiated humic acid at 2.5 kg ha^−1^. Rank stability under alternative weighting scenarios indicates that treatment selection was not dependent on a single weighting assumption. Nevertheless, MCDM should be interpreted as a decision-support summary of the same underlying measurements rather than as independent experimental validation [42–45].

The overall result is a humic substance that is more bioavailable and potent. Its increased hydrophilicity and additional active sites allow gamma-HA to interact more effectively with plant roots or foliage. This may explain the better performance of gamma-irradiated HA observed in the tomato trial. If the irradiated HA outperformed even a higher dose of regular HA, it is plausible—mechanistically—that the irradiated HA can deliver a higher effective concentration of active humic components (despite the same applied dose) because of its increased phenolic content and smaller size. Emerging empirical evidence also supports the enhanced effects of gamma-HA. For instance, agricultural studies have demonstrated that irradiated organic extracts exhibit enhanced bioactivity. One study found that licorice root extract exposed to γ-rays produced a notably higher yield of bioactive compounds, such as volatile oils and glycyrrhizic acid, compared with its non-irradiated counterpart [46,47]. When applied to pear trees, the gamma-irradiated licorice extract outperformed regular humate in boosting fruit yield and quality metrics [48]. Although licorice extract differs from humic acid, these results illustrate the principle that gamma treatments can enhance a bio-stimulant’s efficacy. By analogy, gamma-irradiated HA – with its increased phenolic/carboxylic functionality – can be expected to intensify the positive effects on nutrient uptake, photosynthesis, and stress resistance in tomatoes beyond those achieved by standard HA.

## Conclusions

Moderate gamma irradiation enhanced the biological effectiveness of humic acid in tomato, but the response depended strongly on both irradiation dose and application rate. Among the tested combinations, humic acid irradiated at 2 kGy and applied at 5.0 kg ha^−1^ produced the most consistent improvement in vegetative growth, chlorophyll status, net photosynthetic rate, leaf nutrient status, total soluble phenolics, radical-scavenging capacity, harvest earliness, yield components, marketable yield, and fruit quality. The significant irradiation dose × humic acid rate interaction for marketable yield further demonstrated that irradiation modified the crop response to increasing humic acid input rather than exerting a simple additive effect. In contrast, irradiation at 5 kGy attenuated the benefit of the higher application rate, indicating that greater irradiation intensity did not necessarily improve biostimulant performance.

FTIR analysis showed retention of the principal humic-acid functional-group regions together with dose-dependent spectral changes, supporting physicochemical modification of the material without demonstrating specific bond cleavage or molecular-weight reduction. The agreement among factorial analysis, correlation networks, hierarchical clustering, PCA, and MCDM consistently identified the 2 kGy–5.0 kg ha^−1^ treatment as the best-performing combination under the present greenhouse conditions. However, this dose should be regarded only as the most effective among those tested, not as a universal optimum. Further studies should evaluate a broader irradiation dose range, directly quantify molecular-weight and functional-group changes, assess whole-plant nutrient accumulation, and validate the agronomic and economic performance of the treatment under field conditions.

## Supporting information

S1 Table Humic acid treatments and corresponding application quantities in the tomato pot experiment. This table summarizes the irradiation dose, commercial product rate, active humic acid equivalent, product mass applied per pot and per application, and application method for each experimental treatment.
(DOCX)

S2 Table Post-harvest physicochemical properties of the growing substrate under non-irradiated and gamma-irradiated humic acid treatments. Post-harvest substrate properties include pH, electrical conductivity (EC), organic matter (OM), total nitrogen, nitrate nitrogen (NO_3_–N), available phosphorus, exchangeable potassium, and cation-exchange capacity (CEC).
(DOCX)

S3 Table Principal FTIR bands of non-irradiated and gamma-irradiated humic acid. The table summarizes the principal spectral regions, tentative functional-group assignments, band positions observed at 0, 2, and 5 kGy, and their corresponding interpretations.
(DOCX)

S1 Fig FTIR spectra of non-irradiated and gamma-irradiated humic acid. FTIR spectra are shown for humic acid exposed to 0, 2, and 5 kGy gamma irradiation, illustrating irradiation-associated changes in major absorption regions and spectral features.
(DOCX)

S2 Fig Hybrid weights assigned to the criteria included in the multi-criteria decision-making analysis. Hybrid weights were calculated by equally combining balanced-category weights and CRITIC objective weights.
(DOCX)
