# Supplementary material for: Moderate gamma irradiation enhances the biostimulant activity of humic acid in tomato: Integrated effects on growth, physiology, yield, and fruit quality
Source: PLoS One. 2026 Sep 21;21(9):e0357270. doi: 10.1371/journal.pone.0357270 (PMC13592588; doi:10.1371/journal.pone.0357270)
Supplement: Supplementary file 1 [file pone.0357270.s001.docx]

**Table S1**. Humic acid treatments and corresponding application quantities in the tomato pot experiment

| **Treatment** | **Irradiation dose** | **Commercial product rate** | **Active HA equivalent¹** | **Product mass per pot²** | **Product mass per application³** | **Application method** |
| --- | --- | --- | --- | --- | --- | --- |
| Untreated control | Not applicable | 0 kg ha⁻¹ | 0 kg ha⁻¹ | 0 mg | 0 mg | Water drench |
| Non-irradiated HA, low rate | 0 kGy | 2.5 kg ha⁻¹ | 1.75 kg ha⁻¹ | 250A_pot_ mg | 62.5A_pot_ mg | Soil drench |
| Non-irradiated HA, high rate | 0 kGy | 5.0 kg ha⁻¹ | 3.50 kg ha⁻¹ | 500A_pot_ mg | 125A_pot_ mg | Soil drench |
| 2 kGy-irradiated HA, low rate | 2 kGy | 2.5 kg ha⁻¹ | 1.75 kg ha⁻¹ | 250A_pot_ mg | 62.5A_pot_ mg | Soil drench |
| 2 kGy-irradiated HA, high rate | 2 kGy | 5.0 kg ha⁻¹ | 3.50 kg ha⁻¹ | 500A_pot_ mg | 125A_pot_ mg | Soil drench |
| 5 kGy-irradiated HA, low rate | 5 kGy | 2.5 kg ha⁻¹ | 1.75 kg ha⁻¹ | 250A_pot_ mg | 62.5A_pot_ mg | Soil drench |
| 5 kGy-irradiated HA, high rate | 5 kGy | 5.0 kg ha⁻¹ | 3.50 kg ha⁻¹ | 500A_pot_ mg | 125A_pot_ mg | Soil drench |
