# Supplementary material for: Moderate gamma irradiation enhances the biostimulant activity of humic acid in tomato: Integrated effects on growth, physiology, yield, and fruit quality
Source: PLoS One. 2026 Sep 21;21(9):e0357270. doi: 10.1371/journal.pone.0357270 (PMC13592588; doi:10.1371/journal.pone.0357270)
Supplement: Supplementary file 2 [file pone.0357270.s002.docx]

**Table S2. P**ost-harvest physicochemical properties of the growing substrate under non-irradiated and gamma-irradiated humic acid treatments

| **Treatment** | **pH** | **EC (dS m⁻¹)** | **OM (%)** | **Total N (%)** | **NO₃–N (mg kg⁻¹)** | **Available P (mg kg⁻¹)** | **Exchangeable K (mg kg⁻¹)** | **CEC (cmol(_c) kg⁻¹)** |
| --- | --- | --- | --- | --- | --- | --- | --- | --- |
| Initial substrate¹ | 7.42 | 1.18 | 3.18 | 0.171 | 36.5 | 24.6 | 238 | 17.2 |
| Untreated control | 7.46 ± 0.04 | 0.92 ± 0.02 | 2.86 ± 0.09 | 0.142 ± 0.004 | 17.0 ± 0.5 | 13.8 ± 0.6 | 156.0 ± 4.6 | 15.80 ± 0.33 |
| Non-irradiated HA, 2.5 kg ha⁻¹ | 7.34 ± 0.04 | 1.01 ± 0.02 | 3.02 ± 0.05 | 0.151 ± 0.004 | 20.5 ± 0.0 | 16.2 ± 0.3 | 170.0 ± 2.1 | 16.40 ± 0.28 |
| Non-irradiated HA, 5.0 kg ha⁻¹ | 7.27 ± 0.01 | 1.08 ± 0.04 | 3.18 ± 0.08 | 0.160 ± 0.003 | 23.0 ± 0.5 | 18.0 ± 1.0 | 186.0 ± 3.1 | 17.00 ± 0.22 |
| 2 kGy-irradiated HA, 2.5 kg ha⁻¹ | 7.25 ± 0.05 | 1.04 ± 0.02 | 3.10 ± 0.02 | 0.157 ± 0.003 | 22.0 ± 0.4 | 17.4 ± 0.3 | 180.0 ± 5.9 | 17.10 ± 0.13 |
| 2 kGy-irradiated HA, 5.0 kg ha⁻¹ | 7.18 ± 0.01 | 1.10 ± 0.01 | 3.27 ± 0.05 | 0.166 ± 0.002 | 24.0 ± 0.6 | 18.6 ± 0.8 | 192.0 ± 6.3 | 17.70 ± 0.23 |
| 5 kGy-irradiated HA, 2.5 kg ha⁻¹ | 7.31 ± 0.03 | 0.98 ± 0.04 | 3.00 ± 0.02 | 0.149 ± 0.001 | 19.5 ± 0.8 | 15.5 ± 0.6 | 166.0 ± 5.2 | 16.30 ± 0.22 |
| 5 kGy-irradiated HA, 5.0 kg ha⁻¹ | 7.24 ± 0.03 | 1.03 ± 0.04 | 3.12 ± 0.08 | 0.155 ± 0.003 | 21.5 ± 0.4 | 17.0 ± 0.2 | 176.0 ± 6.6 | 16.80 ± 0.27 |
