# Supplementary material for: Moderate gamma irradiation enhances the biostimulant activity of humic acid in tomato: Integrated effects on growth, physiology, yield, and fruit quality
Source: PLoS One. 2026 Sep 21;21(9):e0357270. doi: 10.1371/journal.pone.0357270 (PMC13592588; doi:10.1371/journal.pone.0357270)
Supplement: Supplementary file 3 [file pone.0357270.s003.docx]

**Table S3.** Principal FTIR bands of non-irradiated and gamma-irradiated humic acid

| **Spectral region and tentative assignment** | **0 kGy** | **2 kGy** | **5 kGy** | **Interpretation** |
| --- | --- | --- | --- | --- |
| O–H stretching in phenolic, alcoholic and carboxylic groups; possible N–H contribution | 3515.66; 3432.08 | 3440.48 | 3434.57 | Broad region retained; small shifts indicate changes in hydrogen-bonding environment |
| Aliphatic C–H asymmetric and symmetric stretching | Not clearly resolved | 2923.79; 2858.10 | Not clearly resolved | Bands were resolved most clearly after 2 kGy irradiation |
| Weak or unassigned combination-band region | Not reported | 2165.85; 2098.34 | 2165.85; 2098.78 | Not assigned to a specific structural change |
| Aromatic C=C, conjugated C=O, carboxylate and/or absorbed-water contribution | 1638.48 | 1636.53 | 1634.69 | Progressive small shift with increasing irradiation dose |
| Aliphatic C–H deformation and/or symmetric COO⁻ stretching | Not clearly resolved | 1461.27 | 1460.05 | Similar band position after 2 and 5 kGy |
| Phenolic O–H, COO⁻, or aliphatic deformation region | Not clearly resolved | Not clearly resolved | 1387.69 | Additional resolved fingerprint band at 5 kGy |
| C–O and C–O–C stretching in phenolic, alcoholic, ether, or carbohydrate-like structures | Not clearly resolved | 1155.25; 1112.77 | 1155.98; 1109.78 | Oxygen-containing fingerprint bands retained after irradiation |
| Low-wavenumber fingerprint region | 587.82 | 599.00 | 692.51 | Dose-dependent modification in the fingerprint region; assignment remains uncertain |
