# Supplementary material for: Moderate gamma irradiation enhances the biostimulant activity of humic acid in tomato: Integrated effects on growth, physiology, yield, and fruit quality
Source: PLoS One. 2026 Sep 21;21(9):e0357270. doi: 10.1371/journal.pone.0357270 (PMC13592588; doi:10.1371/journal.pone.0357270)
Supplement: Supplementary file 5 [file pone.0357270.s005.docx]

**Figure S2. Hybrid weights assigned to the criteria included in the multi-criteria decision-making analysis.** Hybrid weights were calculated by equally combining balanced-category and CRITIC objective weights.
