# Supplementary material for: Moderate gamma irradiation enhances the biostimulant activity of humic acid in tomato: Integrated effects on growth, physiology, yield, and fruit quality
Source: PLoS One. 2026 Sep 21;21(9):e0357270. doi: 10.1371/journal.pone.0357270 (PMC13592588; doi:10.1371/journal.pone.0357270)
Supplement: Supplementary file 6 [file pone.0357270.s004.docx]

Figure S1. FTIR Spectra Of Non-Irradiated And Gamma-Irradiated Humic Acid
